# Movement Disorders in Toxoplasmosis: A Systematic Review

**DOI:** 10.5334/tohm.1093

**Published:** 2025-09-30

**Authors:** Ravindra Kumar Garg, Shweta Pandey, Manoj Agarwal, Sanjay Singhal

**Affiliations:** 1 Department of Neurology, Era’s Lucknow Medical College & Hospital, Era University, Lucknow, India; 2 Department of Neurology, King George’s Medical University, Lucknow, India; 3 Department of Medicine, Era’s Lucknow Medical College & Hospital, Era University, Lucknow, India; 4 Department of Pulmonary Medicine, Dr. Ram Manohar Lohia Institute of Medical Sciences, Lucknow, India

**Keywords:** Cerebellar syndromes, Chorea, Dystonia, Hemiballismus, Movement disorders, Myoclonus, Toxoplasma gondii

## Abstract

**Background:**

Movement disorders are an uncommon but important manifestation of central nervous system toxoplasmosis. Their phenotypes, lesion patterns, and outcomes have not been systematically characterized.

**Methods:**

A PRISMA-based systematic review identified all published patient-level reports of central nervous system toxoplasmosis with movement disorders. Eligible case reports and case series confirmed infection via serology, neuroimaging, polymerase chain reaction, or histopathology. Extracted data included demographics, immune status, clinical and neuroimaging features, lesion location, movement disorder type, treatment, outcomes, and proposed mechanisms. Cases were classified as hyperkinetic, hypokinetic, or cerebellar/ataxia syndromes and summarized descriptively.

**Results:**

Sixty patients were included: hyperkinetic (n = 42), hypokinetic (n = 9), and ataxia/cerebellar (n = 9). HIV-related immunosuppression was the predominant risk factor. Reactivation of latent toxoplasmosis was most frequent (40.5%, 55.6%, 88.9%). Hemichorea/hemiballismus was the leading hyperkinetic phenotype (47.6%), parkinsonism the main hypokinetic form (77.8%), while all ataxia patients exhibited cerebellar signs. Neuroimaging showed basal ganglia involvement in hyperkinetic (47.4%) and hypokinetic (88.9%) cases, whereas ataxia cases consistently demonstrated cerebellar lesions, often with brainstem or hemispheric extension. Standard pyrimethamine–sulfadiazine therapy was used in 59.5%, 33.3%, and 44.4%, respectively. Symptomatic therapy was phenotype-specific: neuroleptics and benzodiazepines for hyperkinetic, levodopa for hypokinetic, and none specified for ataxia. Outcomes varied, with recovery or marked improvement in 57.1%, 33.3%, and 66.7%, and mortality in 33.3%, 33.3%, and 22.2%, respectively. Mechanisms implicated basal ganglia disruption, nigrostriatal degeneration, and cerebellar invasion.

**Conclusions:**

Central nervous system toxoplasmosis-associated movement disorders show distinct phenotype–lesion correlations, with hyperkinetic syndromes most common. Outcomes vary by type, and early recognition with targeted therapy may improve prognosis.

## Introduction

Toxoplasmosis, caused by the protozoan Toxoplasma gondii, is a common parasitic infection. In most individuals with a normal immune system, the infection is either asymptomatic or results in a mild, self-limiting illness. However, in people with weakened immunity, it can cause severe disease of the brain. This is particularly common in those with human immunodeficiency virus infection, advanced acquired immunodeficiency syndrome, recipients of organ transplants, individuals with malignancies, or other conditions that suppress immune function. In such cases, reactivation of a previously dormant infection is the most frequent mechanism. When the central nervous system is involved, toxoplasmosis often presents with headache, seizures, localized neurological deficits, and changes in mental status [1].

Movement disorders are increasingly recognized as important neurological complications in people living with human immunodeficiency virus infection. They can develop at any stage of the illness, from the early symptom-free period to advanced acquired immunodeficiency syndrome, and may present with a wide range of clinical features such as parkinsonism, tremor, chorea, dystonia, myoclonus, and ataxia. These disorders may occur due to opportunistic infections, tumours, medication side effects, or as a component of human immunodeficiency virus–associated neurocognitive and motor syndromes. Their occurrence and pattern vary between populations, influenced by immune function, availability and use of antiretroviral treatment, and the presence of other neurological or systemic diseases [2,3,4]. Presentation of movement disorders may range from hyperkinetic manifestations such as hemichorea, hemiballismus, or choreoathetosis, to insidious hypokinetic syndromes including parkinsonism, or less often, cerebellar ataxia. The type and severity of movement disorder depend largely on the location and extent of brain lesions, with basal ganglia and subthalamic involvement most frequently implicated. These disorders may occur with other focal neurological deficits or cognitive impairment, but can also present as isolated signs. Early recognition is essential, as prompt diagnosis and treatment can lead to significant neurological recovery [3,5].

This systematic review aimed to synthesize all available evidence on movement disorders linked to Toxoplasma gondii infection of the central nervous system and to categorize reported cases into hyperkinetic, hypokinetic, and cerebellar syndromes. It aimed to analyze patient demographics, immune status, modes of infection, and clinical presentations, and to characterize lesion topography and neuroimaging patterns for each movement disorder phenotype. It also sought to summarize anti-toxoplasma treatment regimens, symptomatic management strategies, and clinical outcomes. Finally, our review aimed to providing a comprehensive understanding of their epidemiology, clinical and imaging features, therapeutic approaches, and prognostic implications.

## Methods

This systematic review was done in accordance with the Preferred Reporting Items for Systematic Reviews and Meta-Analyses (PRISMA) guidelines. A pre-defined protocol guided the search strategy, study selection, data extraction, and analysis. The study protocol was registered under the identifier CRD420251119727 [6].

### Search Strategy

A comprehensive search of PubMed, Embase, Scopus, and Google Scholar was conducted from database inception to August 5, 2025. The strategy combined controlled vocabulary (MeSH/Emtree) with free-text keywords, using the following Boolean search string across all databases:

(“Toxoplasmosis” OR “Toxoplasma gondii” OR “toxoplasmic encephalitis”) AND (“movement disorder” OR “parkinsonism” OR “dystonia” OR “chorea” OR “athetosis” OR “ballismus” OR “tremor” OR “myoclonus” OR “ataxia” OR “dyskinesia” OR “bradykinesia” OR “akinesia” OR “rigidity” OR “stereotypy” OR “tic disorder” OR “Tourette syndrome” OR “extrapyramidal”)

Google Scholar was additionally examined for grey literature, and the reference lists of relevant publications were manually reviewed to identify further eligible studies. No language restrictions were imposed during the search; however, only articles providing adequate clinical details were considered for inclusion.

### Eligibility Criteria

**Figure 1:**
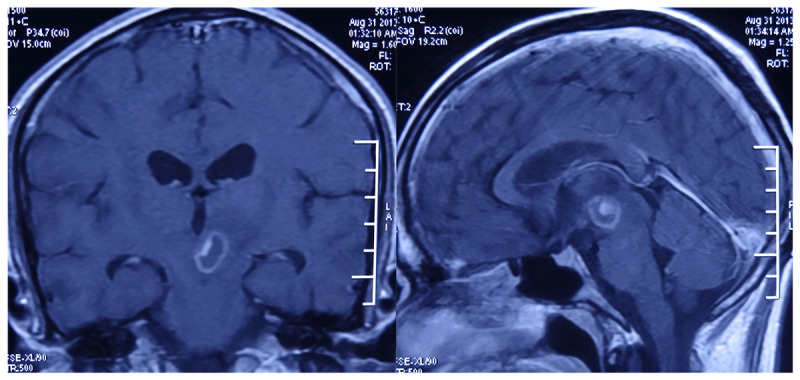
Contrast-enhanced brain MRI in an HIV-positive patient with cerebral toxoplasmosis.

**Figure 2:**
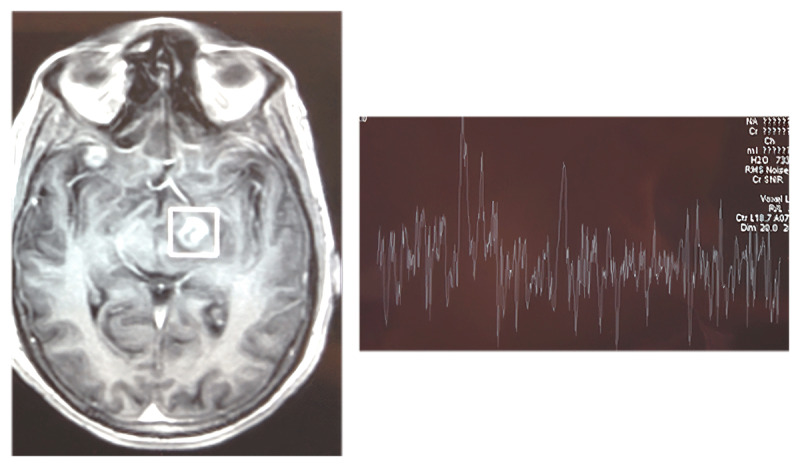
Cerebral toxoplasmosis in an HIV-positive adult — MRI and proton MR spectroscopy. Localized ^1H-MRS acquired from the boxed region demonstrates a prominent lipid–lactate complex with globally reduced metabolites, including decreased N-acetyl aspartate and low choline. The spectral pattern—lipid–lactate prominence without amino-acid peaks. MRS features suggest a necrotizing opportunistic infection and, in the context of HIV, is characteristic of toxoplasmic abscess.

We included published case reports, case series, and observational studies reporting patients of any age with confirmed *Toxoplasma gondii* infection of the central nervous system and a clinically diagnosed movement disorder that occurred in temporal association with the infection. The diagnostic criteria for cerebral toxoplasmosis were as follows:

Proven: Demonstration of *Toxoplasma gondii* tachyzoites in central nervous system tissue or fluid by histology or cytology; if the pattern was unclear, confirmation was obtained by polymerase chain reaction. Probable: Magnetic resonance imaging suggestive of central nervous system toxoplasmosis (multiple or solitary ring-enhancing lesions, often in the basal ganglia, thalamus, or corticomedullary junction, with surrounding edema) and a positive polymerase chain reaction from any central nervous system fluid or tissue, with no evidence of another pathogen. Possible: Magnetic resonance imaging highly suggestive of central nervous system toxoplasmosis (same lesion patterns as above) without laboratory evidence and no evidence of another pathogen; treatment response was not required for inclusion. Infection without central nervous system disease: Positive polymerase chain reaction in blood without evidence of organ involvement; serological testing was excluded [7] (Figure 1 and Figure 2).

Studies without individual patient-level data, animal studies, reviews, editorials, and conference abstracts lacking adequate clinical details were excluded.

### Definitions

Holmes tremor is a slow-frequency tremor, typically below 4.5 hertz, characterized by an irregular pattern combining resting, postural, and action components, most commonly involving the upper extremities [8]. Other subtypes of movement disorders were classified according to the definitions provided by the Movement Disorder Society [9] (Video 1).

**Video 1:** Video of an HIV-positive patient with cerebral toxoplasmosis showing right-sided hemichorea, predominantly involving the lower limb.

### Study Selection

All retrieved references were imported into EndNote 21 (Clarivate Analytics, Philadelphia, United States) for duplicate removal. Title and abstract screening for relevance was performed independently by two reviewers. Full texts of studies deemed potentially eligible were then assessed in detail against the predefined inclusion criteria. Any discrepancies were resolved through discussion or, when necessary, consultation with a third reviewer. The study selection process was recorded in accordance with PRISMA guidelines and presented in a flow diagram.

### Data Extraction

A standardized data extraction form was developed and piloted. Two reviewers independently extracted the following variables: author, year, country, patient age and sex, immune status, CD4 count (if human immunodeficiency virus positive), duration of illness, mode of infection, diagnostic method, central nervous system involvement pattern, lesion location and neuroimaging features, movement disorder type, onset relative to toxoplasmosis diagnosis, duration of movement disorder, additional neurological features, anti-toxoplasma treatment regimen and duration, symptomatic therapy, clinical outcome, follow-up duration, and proposed pathogenic mechanism.

### Risk of Bias Assessment

The quality of the included case reports and case series was evaluated using the assessment tool developed by Murad et al., which examines four domains: selection, ascertainment, causality, and reporting [10]. Each study was assessed and rated as “good,” “fair,” or “poor” based on the number of domains adequately fulfilled. Evaluations were conducted independently by two reviewers, with any discrepancies resolved through discussion until consensus was achieved [11].

### Data Synthesis

Extracted data were analyzed using descriptive statistics. Categorical variables were reported as frequencies and percentages, while continuous variables were presented as mean, median, range, and interquartile range, as appropriate. Cases were grouped into three movement disorder categories, like, hyperkinetic, hypokinetic, and ataxia/cerebellar syndromes. Lesion distribution patterns, neuroimaging findings, and associated clinical features were described for each group.

## Results

**Figure 3:**
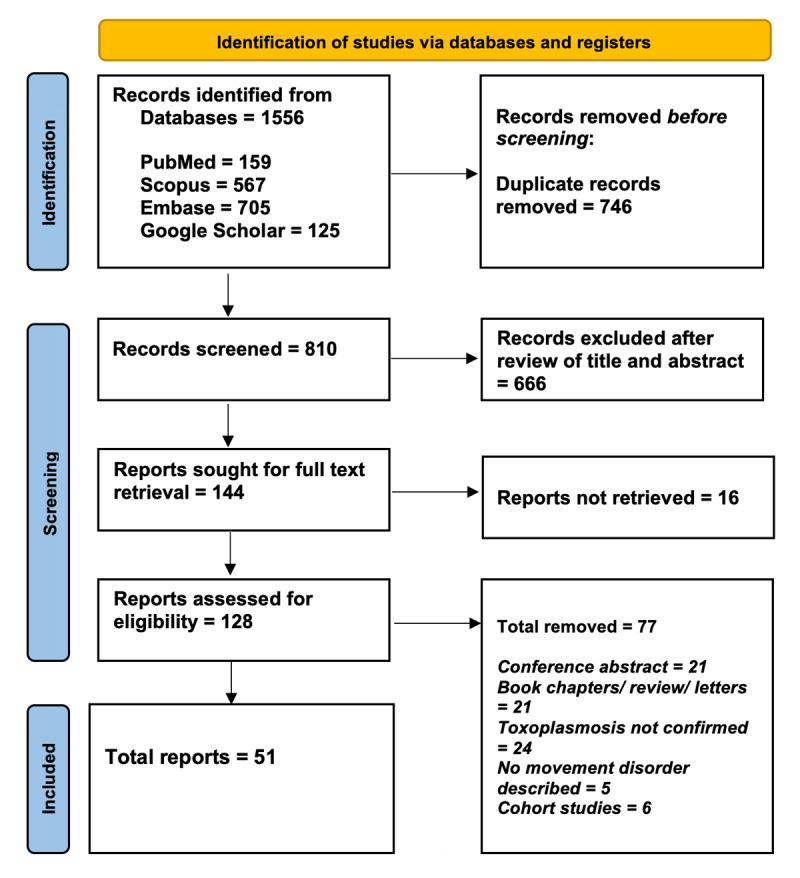
PRISMA flow diagram showing the process of study identification, screening, eligibility assessment, and final inclusion.

The systematic review analyzed 60 patients with movement disorders secondary to toxoplasmosis, categorized into hyperkinetic disorders (n = 42), hypokinetic disorders (n = 9), and ataxia or other cerebellar syndromes (n = 9) (Supplementary Table 1, Supplementary Table 2 and Supplementary Table 3). All individual cases were assessed as being of good quality (Supplementary Item 1). The PRISMA checklist of the review is available in Supplementary Item 2. The PRISMA flow diagram illustrating record selection process has been provided as Figure 3. Patients were classified according to the predominant syndrome like hyperkinetic, hypokinetic, or ataxic, though occasional cases demonstrated overlapping or additional movement disorder features.

The mean age was 38.7 years for hyperkinetic, 52.9 years for hypokinetic, and 45.1 years for ataxia patients, with overall ranges from 11 to 78 years. Males predominated in the hyperkinetic group (78.6%) while sex distribution was more balanced in hypokinetic (55.6% males) and ataxia (55.6% males) cases (Table 1).

**Table 1:** Comparative Clinical, Demographic, Neuroimaging, and Outcome Characteristics of Hyperkinetic, Hypokinetic, and Cerebellar Movement Disorders in Central Nervous System Toxoplasmosis (Summary of Supplementary table 1–3).


VARIABLE	HYPERKINETIC MOVEMENT DISORDERS (N: 42)	HYPOKINETIC MOVEMENT DISORDERS (N: 9)	ATAXIA AND OTHER CEREBELLAR SYNDROMES (N: 9)

**Age (in years)**	Mean: 38.7 Median: 36.5 Mode: 35 Range: 11–78 IQR: 11	Mean: 52.9 Median: 56 Range: 31–66 IQR: 16 NA: 1	Mean: 45.1 Median: 50 Mode: 53 Range: 23–60 IQR: 18.5

**Sex**	Male (M): 33 (78.6%) Female (F): 9 (21.4%)	Male: 5 (55.6%) Female: 4 (44.4%)	Female (F): 4 (44.4%) Male (M): 5 (55.6%)

**Geographic distribution of published reports**	Total: 33 Africa: 2 (6.1%) Asia: 3 (9.1%) Europe: 8 (24.2%) North America: 12 (36.4%) South America: 8 (24.2%)	Total: 9 Asia: 2 (22.2%) Europe: 4 (44.4%) North America: 3 (33.3%)	Total: 9 Asia – 4 (44.4%) Europe – 3 (33.3%) North America – 2 (22.2%)

**Immune status**	HIV-related immunosuppression: 37 (88.1%) Non-HIV immunocompromised: 1 (2.4%) Immunocompetent: 4 (9.5%)	HIV-positive (including all with AIDS stage, ART, well-controlled, confirmed by tests): 7 (77.8%) Immunocompetent (HIV-negative): 1 (11.1%) Other immunocompromised (non-HIV): 1 (11.1%)	HIV-positive/AIDS: 6 (66.7%) Other immunocompromised (non-HIV): 1 (11.1%) Immunocompetent: 2 (22.2%)

**CD4 count**	<50 cells/µL: 12 (28.6%) 50–99 cells/µL: 6 (14.3%) 100–199 cells/µL: 3 (7.1%) ≥200 cells/µL: 2 (4.8%) Other specified values: 2 (4.8%) Not applicable/not reported: 17 (40.5%)	<50 cells/µL: 2 (22.2%) 100–199 cells/µL: 2 (22.2%) ≥200 cells/µL: 3 (33.3%) Not applicable/not reported: 2 (22.2%)	<50 cells/µL: 2 (22.2%) 50–99 cells/µL: 1 (11.1%) 100–199 cells/µL: 1 (11.1%) Not applicable/not reported: 5 (55.6%)

**Duration of illness**	<2 weeks: 8 (21.1%) 2 weeks–<3 months: 13 (34.2%) ≥3 months: 10 (26.3%) Not specified/unknown: 7 (18.4%)	<2 weeks: 2 (22.2%) 2 weeks–<3 months: 2 (22.2%) ≥3 months: 5 (55.6%)	<2 weeks: 4 (44.4%) 2 weeks–<3 months: 4 (44.4%) ≥3 months: 1 (11.1%)

**Likely Mode of Infection**	Reactivation of latent toxoplasmosis: 17 (40.5%) Oral ingestion of oocysts: 3 (7.1%) Vertical transmission: 1 (2.4%) Blood transfusion: 1 (2.4%) Unknown/not specified: 20 (47.6%)	Reactivation of latent toxoplasmosis: 5 (55.6%) Hematogenous dissemination: 1 (11.1%) Intravenous drug use: 1 (11.1%) Blood transfusion: 1 (11.1%) Unknown/not specified: 1 (11.1%)	Reactivation/likely reactivation: 8 (88.9%) Acquired (new infection): 1 (11.1%)

**Diagnostic Method for Toxoplasmosis**	Serology + Neuroimaging: 19 (45.2%) Serology + Neuroimaging + Clinical response: 6 (14.3%) PCR-based: 4 (9.5%) Histopathology/Autopsy: 3 (7.1%) Neuroimaging only: 1 (2.4%) Other/mixed methods: 9 (21.4%)	MRI ± serology (without DaTSCAN): 3 (33.3%) DaTSCAN (with MRI + serology): 1 (11.1%) Molecular/serological confirmation (PCR or high IgG titers): 3 (33.3%) Histopathology/biopsy confirmation: 1 (11.1%) Presumed (epidemiologic evidence only): 1 (11.1%)	Serology + Neuroimaging: 3 (33.3%) Serology + Neuroimaging + Histopathology: 2 (22.2%) PCR-based (CSF/blood/tissue): 2 (22.2%) Histopathology only (with or without serology): 1 (11.1%) Other/specialized tests (e.g., Sabin-Feldman dye test, mouse inoculation): 1 (11.1%)

**CNS Involvement Pattern**	Basal ganglia involvement: 18 (47.4%) Thalamic involvement: 14 (36.8%) Midbrain/brainstem involvement: 8 (21.1%) Cortical involvement: 8 (21.1%) Cerebellar involvement: 3 (7.9%) Diffuse/widespread: 2 (5.3%) White matter involvement: 2 (5.3%) Subthalamic nucleus involvement: 2 (5.3%) Normal neuroimaging: 1 (2.6%)	Basal ganglia involvement (with or without other regions): 6 (66.7%) Thalamic involvement (with or without other regions): 2 (22.2%) Midbrain/brainstem involvement: 2 (22.2%) Cortical involvement (frontal, parietal, occipital, temporo-occipital): 3 (33.3%) Diffuse/widespread involvement: 1 (11.1%) White matter involvement: 1 (11.1%)	Cerebellar involvement (with or without other regions): 9 (100%) Brainstem involvement: 2 (22.2%) Thalamic involvement: 1 (11.1%) Cerebral hemispheres involvement: 2 (22.2%) Spinal cord/nerve root involvement: 1 (11.1%) Leptomeningeal involvement: 1 (11.1%)

**Dominant movement disorders present**	Hemichorea/Hemiballismus/Hemichorea-hemiballismus/Hemichoreoathetosis/Choreoballism: 20 (47.6%) Holmes tremor/Rubral tremor: 7 (16.7%) Chorea (generalized/focal): 5 (11.9%) Myoclonus: 2 (4.8%) Dyskinesia: 1 (2.4%) Tic disorder: 1 (2.4%) Akathisia: 1 (2.4%) Mixed chorea + dystonia + parkinsonism: 1 (2.4%) Dystonia (segmental/hemidystonia/focal): 3 (7.1%) Other types of tremor (non-Holmes): 1 (2.4%)	Hemiparkinsonism: 3 (33.3%) Bilateral parkinsonism: 4 (44.4%) Mixed/extrapyramidal + other features: 1 (11.1%) Subacute parkinsonism: 1 (11.1%)	Ataxia (with or without other cerebellar signs): 9 (100%) Dysarthria: 3 (33.3%) Nystagmus: 2 (22.2%) Gait disturbance/wide-based gait: 2 (22.2%) Dysmetria/incoordination: 2 (22.2%) Intention tremor: 1 (11.1%)

**Onset relative to toxoplasmosis**	At initial presentation/concurrent with diagnosis: 15 (35.7%) Delayed onset (weeks–months): 12 (28.6%) Long-term onset (≥1 year): 5 (11.9%) During disease progression/relapse/advanced stage: 6 (14.3%) Treatment-related/iatrogenic (new group): 2 (4.8%) Not clearly specified: 2 (4.8%)	At initial presentation/concurrent with diagnosis: 2 (22.2%) Delayed onset (weeks to months after diagnosis/treatment): 4 (44.4%) Long-term onset (≥1 year after diagnosis): 3 (33.3%)	At initial presentation/concurrent with diagnosis: 6 (66.7%) Delayed onset (weeks to months after diagnosis/treatment): 2 (22.2%) During disease progression/advanced stage: 1 (11.1%)

**Duration of movement disorder/Ataxia**	<2 weeks: 7 (16.7%) 2 weeks–<3 months: 16 (38.1%) ≥3 months–<1 year: 6 (14.3%) Long-term/persistent (≥1 year or until death): 13 (31.0%)	2 weeks–<3 months: 0 (0%) ≥3 months–<1 year: 4 (44.4%) Long-term/persistent (≥1 year or until death): 5 (55.6%)	≥3 months–<1 year: 4 (44.4%) Long-term/persistent (≥1 year or until death): 5 (55.6%)

**Other neurological features**	Motor deficits: 19 (50%) Cognitive/behavioral changes: 15 (35.7%) Speech disorders: 9 (21.4%) Cranial nerve palsies: 4 (9.5%) Visual disturbances: 3 (7.1%) Seizures: 3 (7.1%) Cerebellar signs: 3 (7.1%) Signs of raised intracranial pressure: 3 (7.1%) No additional neurological findings: 3 (7.1%) Systemic/secondary (new group): 2 (4.8%)	Cognitive impairment/decline: 4 (44.4%) Motor deficits: 3 (33.3%) Behavioral/psychiatric changes: 2 (22.2%) Cranial nerve involvement: 2 (22.2%) Other neurological signs: 3 (33.3%)	Headache-related: 4 (44.4%) Cranial nerve palsy: 1 (11.1%) Motor weakness: 2 (22.2%) Consciousness/cognitive impairment: 3 (33.3%) Peripheral nerve signs: 3 (33.3%) Other focal signs: 1 (11.1%)

**Neuroimaging features**	Ring-enhancing/granulomatous lesions: 31 (73.8%) Non-enhancing hyperintense/hypointense lesions: 4 (9.5%) Diffuse atrophy/edema (non-focal): 3 (7.1%) Normal imaging: 2 (4.8%) Unspecified/Other CT-MRI: 2 (4.8%)	Ring-enhancing lesions (including abscesses) on MRI/CT: 4 (44.4%) Gliosis/encephalomalacia: 2 (22.2%) White matter changes/atrophy: 1 (11.1%) Characteristic/atypical (“bullseye”) lesions: 1 (11.1%) Small enhancing internal capsule lesions: 1 (11.1%)	Cerebellar involvement: 3 (33.3%) Thalamic/cerebellitis/muscle involvement: 1 (11.1%) Basal ganglia ± multiple sites: 1 (11.1%) Cerebral hemispheres ± herniation: 1 (11.1%) Post-operative change/progression: 1 (11.1%) Normal imaging: 1 (11.1%)

**Lesion Location**	*(multiple sites possible per patient)* Basal ganglia: 23 (54.8%) Thalamus: 18 (42.9%) Subthalamic nucleus: 10 (23.8%) Midbrain/brainstem: 9 (21.4%) Frontal lobe: 9 (21.4%) Parietal lobe: 5 (11.9%) Occipital lobe: 4 (9.5%) Temporal lobe: 3 (7.1%) Cerebellum: 3 (7.1%) White matter: 3 (7.1%) Normal/no lesion: 2 (4.8%) Diffuse/meningitis-related: 1 (2.4%)	Basal ganglia: 8 (88.9%) Thalamus: 3 (33.3%) Midbrain/brainstem: 1 (11.1%) Frontal lobe: 2 (22.2%) Parietal lobe: 1 (11.1%) White matter involvement: 3 (33.3%) Corpus callosum: 1 (11.1%)	Cerebellum only: 3 (33.3%) Cerebellum + other CNS sites: 4 (44.4%) Non-cerebellar CNS sites only: 1 (11.1%) Muscle ± spinal: 1 (11.1%)

**Treatment**	Standard anti-toxoplasma regimen (pyrimethamine + sulfadiazine ± folinic acid/leucovorin ± steroids): 25 (59.5%) Alternative regimens (TMP-SMX, clindamycin, spiramycin, other sulfa drugs, pyrimethamine monotherapy): 10 (23.8%) Empirical/unspecified therapy: 2 (4.8%) No treatment/not applicable: 5 (11.9%)	Pyrimethamine + sulfadiazine (with or without folinic acid): 3 (33.3%) Pyrimethamine + other agents (clindamycin, TMP-SMX, corticosteroids): 3 (33.3%) Trimethoprim-sulfamethoxazole (TMP-SMX) + ART/HAART: 1 (11.1%) No treatment given/diagnosis post-mortem: 1 (11.1%) Not reported: 1 (11.1%)	Pyrimethamine + sulfadiazine ± folinic acid/leucovorin ± ART ± corticosteroids: 4 (44.4%) Pyrimethamine + sulfadiazine (induction) + corticosteroids + mycophenolate mofetil: 1 (11.1%) Clindamycin-based regimen (± pyrimethamine, azithromycin, pyridoxine): 2 (22.2%) IV cotrimoxazole (± corticosteroids, ART): 1 (11.1%) No specific anti-Toxoplasma therapy (empirical antibiotics only): 1 (11.1%)

**Duration of Treatment**	<1 month: 4 (9.5%) 1 month–<3 months: 9 (21.4%) ≥3 months–<1 year: 5 (11.9%) ≥1 year/long-term: 3 (7.1%) Not specified/not stated: 19 (45.2%) Mixed/multiple-course therapy: 2 (4.8%)	<2 months: 3 (33.3%) 2–<6 months: 2 (22.2%) ≥6 months: 1 (11.1%) Not specified/not applicable: 3 (33.3%)	< 2 weeks: 3 (33.3%) 2–8 weeks: 3 (33.3%) > 8 weeks/multiple courses: 2 (22.2%) Not applicable: 1 (11.1%)

**Symptomatic Therapy for movement disorders**	Neuroleptics (haloperidol, risperidone, thioridazine, olanzapine, quetiapine, aripiprazole): 14 (33.3%) VMAT inhibitors (tetrabenazine): 3 (7.1%) Benzodiazepines (clonazepam, diazepam, delorazepam): 9 (21.4%) Dopaminergic/Parkinsonian drugs (levodopa, amantadine, propranolol): 6 (14.3%) Anticonvulsants (valproate, phenytoin, carbamazepine, oxcarbazepine, primidone, levetiracetam): 6 (14.3%) Anticholinergics (trihexyphenidyl): 4 (9.5%) Botulinum toxin/splints: 3 (7.1%) Muscle relaxant (baclofen): 1 (2.4%) Other (isoniazid, dexamethasone): 2 (4.8%) No treatment/not given/not specified: 12 (28.6%)	Levodopa/levodopa-carbidopa: 5 (55.6%) Benzodiazepines/muscle relaxants: 1 (11.1%) Antipsychotics (risperidone trial): 1 (11.1%) None specific (improved with antiparasitic/ART only): 1 (11.1%) Not specified/not applicable: 1 (11.1%)	Not specified: 9 (100%)

**Outcome**	Complete recovery: 10 (23.8%) Marked/partial improvement (residual symptoms): 14 (33.3%) No significant improvement: 2 (4.8%) Death (any cause): 14 (33.3%) Other specific outcomes (AZT/radiotherapy): 2 (4.8%)	Persistent symptoms (including parkinsonism/dystonia): 3 (33.3%) Marked or complete recovery: 1 (11.1%) Partial improvement: 2 (22.2%) Death: 3 (33.3%)	Improvement/remission: 6 (66.7%) Fatal outcome: 2 (22.2%) Relapse/residual deficits: 1 (11.1%)

**Follow-up duration**	<3 months: 6 (14.3%) 3–<6 months: 8 (19.0%) 6–<12 months: 2 (4.8%) ≥1 year: 6 (14.3%) Until death: 10 (23.8%) Not specified/not stated/none: 10 (23.8%)	<6 months: 3 (33.3%) 6 months–<1 year: 2 (22.2%) 1–<3 years: 1 (11.1%) ≥3 years: 1 (11.1%) Not reported/unclear/post-mortem: 2 (22.2%)	< 6 months: 5 (55.6%) ≥ 6 months: 3 (33.3%) No follow-up: 1 (11.1%)

**Proposed Pathogenic Mechanisms**	Basal ganglia lesions/circuit disruption (including subthalamic, pallidal, thalamic involvement): 19 (45.2%) Cerebellar–thalamo–cortical/dentato–rubro–olivary pathway disruption: 6 (14.3%) Midbrain/red nucleus involvement (Holmes tremor, rubral tremor): 4 (9.5%) Diffuse/bilateral subcortical involvement (generalized movement disorders): 3 (7.1%) HIV encephalopathy/combined pathology (HIV + toxoplasmosis ± other lesions): 3 (7.1%) Functional/mixed organic–functional contribution: 1 (2.4%) Iatrogenic/catecholamine-induced: 1 (2.4%) Other specific (subcortical myoclonus, brainstem involvement without basal ganglia lesion): 1 (2.4%) Unclassified/not clearly stated in reports: 4 (9.5%	Basal ganglia lesions disrupting dopaminergic/motor circuits: 5 (55.6%) Post-infectious degeneration of nigrostriatal pathway: 2 (22.2%) Combined basal ganglia and thalamic circuit involvement: 1 (11.1%) Necrotizing toxoplasma encephalitis (glioma mimic) in immunocompetent: 1 (11.1%)	Reactivation – immunosuppression: 5 (55.6%) Direct invasion/neurotropism: 3 (33.3%) Immune defect (G6PD): 1 (11.1%)


AIDS: Acquired Immunodeficiency Syndrome; ART: Antiretroviral Therapy; CD: Cluster of Differentiation; CNS: Central Nervous System; CSF: Cerebrospinal Fluid; F: Female; G6PD: Glucose-6-Phosphate Dehydrogenase; HAART: Highly Active Antiretroviral Therapy; HIV: Human Immunodeficiency Virus; IQR: Interquartile Range; IV: Intravenous; M: Male; MRI: Magnetic Resonance Imaging; NA: Not Available/Not Applicable; PCR: Polymerase Chain Reaction; TMP-SMX: Trimethoprim–Sulfamethoxazole; VMAT: Vesicular Monoamine Transporter.

Immune status differed across groups: HIV-related immunosuppression predominated in hyperkinetic cases (88.1%), was slightly lower in hypokinetic cases (77.8%), and least frequent in ataxia (66.7%), with the remainder split between other immunocompromised states and immunocompetent individuals. CD4 counts reflected this pattern, with severe depletion (<50 cells/µL) in 28.6% of hyperkinetic, 22.2% of hypokinetic, and 22.2% of ataxia patients, while higher counts (≥200 cells/µL) were more common in hypokinetic (33.3%) than in hyperkinetic (4.8%) or ataxia (not reported in over half). Illness duration also varied: hyperkinetic cases most often presented subacutely (34.2% between 2 weeks and <3 months), hypokinetic cases had a chronic course in more than half (55.6%), whereas ataxia was characterized by acute or subacute presentations (44.4% each). The likely mode of infection was reactivation of latent toxoplasmosis in 40.5% of hyperkinetic, 55.6% of hypokinetic, and 88.9% of ataxia cases, with ingestion, transfusion, or intravenous drug use reported rarely. Diagnostic approaches showed similar trends, with serology plus neuroimaging most frequent in hyperkinetic (45.2%) and also common in ataxia (33.3%), while hypokinetic cases more often required advanced confirmation, including molecular assays or DaTSCAN (Table 1).

The movement disorder spectrum was dominated by hemichorea and related hyperkinetic syndromes in 47.6% of hyperkinetic patients, followed by Holmes or rubral tremor (16.7%), generalized chorea (11.9%), and less frequent entities including dystonia, myoclonus, tics, dyskinesia, or akathisia. Hypokinetic cases were largely characterized by parkinsonism, with bilateral features in 44.4%, hemiparkinsonism in 33.3%, and mixed or subacute forms in a minority. Ataxia cases uniformly demonstrated cerebellar involvement, often accompanied by dysarthria (33.3%), nystagmus (22.2%), dysmetria (22.2%), or gait disturbance. In the hyperkinetic group, one patient (1/42; 2.4%) exhibited a combination of chorea, dystonia, and parkinsonism. Similarly, within the hypokinetic group, one case (1/9; 11.1%) presented with hemiparkinsonism on one side and contralateral dystonia/chorea. No mixed patterns were recorded among the ataxia/cerebellar cases (Table 1).

Central nervous system lesion patterns paralleled these phenotypes. Hyperkinetic disorders showed predominant basal ganglia involvement (47.4%) along with thalamic (36.8%), midbrain/brainstem (21.1%), cortical (21.1%), and occasional cerebellar or diffuse lesions. Hypokinetic cases had even stronger basal ganglia localization (66.7%), frequently with thalamic (22.2%) or cortical (33.3%) pathology. Ataxia was consistently linked to cerebellar lesions (100%), with nearly half showing additional sites such as brainstem, hemispheres, thalamus, spinal cord, or leptomeninges. Additional clinical features also differed. Hyperkinetic patients frequently exhibited motor deficits (50%) and cognitive or behavioral symptoms (39.5%), hypokinetic patients often developed cognitive decline (46.2%) and motor impairment (38.5%), while ataxia cases were marked by headache (44.4%), cognitive changes (33.3%), and peripheral nerve involvement (33.3%) (Table 1).

Neuroimaging revealed distinct patterns across groups. In hyperkinetic cases, the hallmark finding was ring-enhancing or granulomatous lesions (73.8%), while a smaller proportion showed non-enhancing hyperintense or hypointense lesions (9.5%), diffuse atrophy or edema (7.1%), or normal scans (4.8%). Hypokinetic cases also commonly displayed ring-enhancing lesions (44.4%), but gliosis or encephalomalacia (22.2%), white matter changes or atrophy (11.1%), and atypical “bullseye” lesions (11.1%) were more characteristic, with one case localized to the internal capsule. In ataxia, imaging reflected the underlying cerebellar pathology, with isolated cerebellar lesions in 33.3% and combined cerebellar plus other central nervous system sites in 44.4%, alongside occasional thalamic, hemispheric, or muscle involvement; one case showed post-operative progression and another normal imaging (Table 1).

Lesion distribution also varied: basal ganglia (54.8%) and thalamus (42.9%) predominated in hyperkinetic cases, with additional subthalamic nucleus, brainstem, and cortical sites. Hypokinetic cases showed strong basal ganglia localization (88.9%), often with white matter or frontal lobe changes. In contrast, ataxia was consistently linked to cerebellar lesions, either isolated or in combination with other sites (Table 1).

Standard anti-toxoplasma therapy with pyrimethamine plus sulfadiazine (± folinic acid and corticosteroids) was most frequently used, given in 25 patients (59.5%) with hyperkinetic disorders, 3 patients (33.3%) with hypokinetic features, and 4 patients (44.4%) with ataxia. Alternative regimens such as clindamycin- or cotrimoxazole-based therapy were reported in 10 hyperkinetic (23.8%), 3 hypokinetic (33.3%), and 3 ataxia patients (33.3%). Treatment duration varied: hyperkinetic cases often received 1–3 months (21.4%) or long-term therapy (31.0% ≥3 months), hypokinetic cases were treated for 2–6 months (22.2%) or longer (11.1%), while ataxia patients more frequently had short courses, with 6 of 9 cases (66.6%) treated ≤8 weeks. Symptomatic therapy was common in hyperkinetic cases, with neuroleptics used in 14 patients (33.3%), benzodiazepines in 9 (21.4%), dopaminergic drugs in 6 (14.3%), and anticonvulsants in 6 (14.3%). In contrast, hypokinetic patients mainly received levodopa-based treatment (5; 55.6%), while symptomatic management was not specified for ataxia cases (100%) (Table 1).

Proposed pathogenic mechanisms varied across movement disorder phenotypes but consistently highlighted circuit-level disruptions. In hyperkinetic cases, basal ganglia involvement was the dominant mechanism (45.2%), often extending to subthalamic, pallidal, or thalamic regions. Other pathways implicated included cerebellar–thalamo–cortical and dentato–rubro–olivary circuits (14.3%), as well as midbrain and red nucleus lesions associated with Holmes tremor (9.5%). A minority reflected diffuse subcortical involvement (7.1%), HIV encephalopathy with combined pathology (7.1%), or rare mechanisms such as functional contributions and iatrogenic catecholamine-induced syndromes. Hypokinetic cases showed even stronger localization, with dopaminergic basal ganglia circuit disruption in 55.6%, post-infectious nigrostriatal degeneration in 22.2%, and combined basal ganglia–thalamic circuit lesions in 11.1%, alongside a striking report of necrotizing toxoplasma encephalitis mimicking glioma in an immunocompetent patient. Ataxia cases were attributed to broader mechanisms, with reactivation under immunosuppression predominating (55.6%), but direct neurotropic invasion of cerebellar structures (33.3%) and a rare immune defect (G6PD deficiency, 11.1%) also contributing (Table 1).

## Discussion

Our review describes the clinical, neuroimaging, and mechanistic patterns of movement disorders in central nervous system toxoplasmosis, categorized as hyperkinetic, hypokinetic, or cerebellar. Hyperkinetic syndromes, including hemichorea, hemiballismus, and choreoathetosis, formed the largest group and were linked to contralateral basal ganglia or subthalamic nucleus lesions, indicating basal ganglia–thalamocortical circuit disruption and the organism’s affinity for deep grey matter. These disorders often began abruptly, and the younger mean age in this group likely reflects HIV-associated acute basal ganglia toxoplasmosis in younger adults. By contrast, cerebellar syndromes were least frequent, showing cerebellar lesions with occasional brainstem or hemispheric extension that produced dysarthria, nystagmus, and dysmetria.

Hypokinetic syndromes, mainly parkinsonism, were the next most common and presented with bradykinesia, rigidity, and gait disturbance, while tremor was uncommon and milder than in idiopathic Parkinson’s disease. They generally emerged late, progressed slowly, and were often associated with bilateral basal ganglia lesions, suggesting post-infectious or degenerative injury to the nigrostriatal pathway. The poor levodopa response and persistent deficits support an irreversible structural basis rather than a reversible dopaminergic deficiency, although drug-induced parkinsonism may have contributed in isolated cases. Standard therapy included pyrimethamine, sulfadiazine, corticosteroids, and antiretroviral drugs, yet many patients had long lasting deficits. Importantly, host immune status influenced presentation: immunosuppressed patients typically exhibited acute basal ganglia lesions with severe hyperkinetic or mixed syndromes, whereas immunocompetent individuals more often developed delayed or atypical phenotypes from secondary circuit degeneration, underscoring distinct mechanisms and outcomes across immune states.

The mechanism of parkinsonism in cerebral toxoplasmosis remains unclear, as many patients lacked active lesions, showed limited response to dopaminergic therapy, and had poor outcomes. The syndrome is likely multifactorial, driven by post-infectious nigrostriatal degeneration, comorbid HIV-related immune dysfunction, and potential drug-induced effects. More broadly, the pathogenesis of movement disorders in toxoplasmosis appears to involve a combination of direct parasite-mediated injury, vascular compromise, neurotransmitter dysregulation, and secondary neurodegenerative changes. Structural lesions most often occur in the basal ganglia, thalamus, and subthalamus, where tachyzoite proliferation within neurons and glial cells causes necrotizing damage, disrupting motor control pathways. Perilesional inflammation, vasculitis, thrombosis, and edema further exacerbate injury by inducing ischemia. Neuronal infection with tissue cysts has been shown to alter extracellular vesicle release and composition, incorporating parasite antigens such as dense granule proteins, which stimulate inflammatory gene expression in astrocytes and suppress glutamate transporter 1, impairing glutamate clearance and disrupting excitatory neurotransmission in basal ganglia circuits, leading to hyperkinetic syndromes and excitotoxic damage to dopaminergic neurons [12]. Acute infection can also activate brain endothelial cells, producing platelet–fibrin microthrombi that impair cerebral blood flow; anticoagulant therapy may improve perfusion without reducing parasite load, suggesting ischemia as an independent cause of neurological dysfunction [13]. Infection has been associated with increased tyrosine hydroxylase expression, enhancing dopamine synthesis, while reducing dopamine D1 receptor, dopamine transporter, and Nurr1 expression, indicating impaired receptor signaling and reuptake [14]. *Toxoplasma gondii* also induces neuroimmune responses distinct from those caused by other neurotropic pathogens, such as West Nile virus and Zika virus, suggesting potential for pathogen-specific therapeutic strategies [15]. These mechanisms align with clinical observations, where hyperkinetic disorders are linked to basal ganglia and subthalamic lesions, hypokinetic syndromes to nigrostriatal degeneration, and cerebellar syndromes to dentate nucleus and superior cerebellar peduncle involvement. While immunosuppression, particularly from human immunodeficiency virus, is the strongest risk factor, cases also occur in immunocompetent individuals.

*Toxoplasma gondii* has been associated with several other neurological disorders, including Alzheimer’s disease, Parkinson’s disease, essential tremor, and other movement disorders. A systematic review and meta-analysis of thirty-four observational studies involving more than twenty-four thousand participants reported a significant association between *Toxoplasma gondii* infection and Alzheimer’s disease, with impairments in global cognition, verbal fluency, and memory [16]. Conversely, evidence for Parkinson’s disease remains inconsistent, as prior meta-analyses and molecular studies reported null or variable findings [17,18,19]. In essential tremor, case–control studies revealed markedly higher anti-*Toxoplasma gondii* immunoglobulin G seropositivity in patients than controls (43.4% vs. 12%) [20]. Notably, experimental Parkinson’s disease models indicate potential neuroprotective effects of *Toxoplasma gondii* infection, including improved motor function, reduced catalepsy, altered nociception, enhanced oxidative balance, and elevated striatal dopamine and brain-derived neurotrophic factor [21].

On neuroimaging, while ring-enhancing or granulomatous lesions predominated in hyperkinetic patients (81.6%), they were less frequent in hypokinetic cases (69.2%), where parkinsonism often reflected post-infectious nigrostriatal degeneration, gliosis, or even drug-related effects, highlighting that hypokinetic syndromes may arise without direct correlation to active lesion burden. All patients with ataxia had imaging abnormalities, with over half showing multifocal involvement of cerebellar and extra-cerebellar structures rather than isolated cerebellar lesions. In the hypokinetic group, functional imaging was available in two isolated cases. DAT and DaTSCAN studies in these cases demonstrated striatal dopaminergic deficits, with reduced putaminal uptake corresponding to lesions in the thalamus and basal ganglia. Importantly, these findings occurred in the absence of α-synuclein pathology, as confirmed by ancillary testing in one patient, supporting a non-synucleinopathic, post-infectious mechanism. Such results indicate that parkinsonism in cerebral toxoplasmosis arises from structural and functional disruption of the nigrostriatal pathway rather than from a primary degenerative process [22,23].

This review has several limitations. Most available data come from isolated case reports or small series, limiting generalizability and precluding robust statistical analysis. Publication bias is likely, as unusual or severe presentations are more often reported. Considerable heterogeneity exists in diagnostic criteria, neuroimaging protocols, and outcome measures, making uniform comparisons difficult. Long-term follow-up is rarely available, restricting insights into prognosis. Coexisting neurological disorders, particularly in individuals with human immunodeficiency virus infection, may confound attribution of movement disorders solely to toxoplasmosis. The observational nature of included studies further limits the ability to establish causal links between lesion location and phenotype. Another important gap is the scarcity of functional imaging. Although a few hypokinetic cases had DaTSCAN evidence of striatal dopaminergic deficits without α-synuclein pathology, no FDOPA PET studies were reported. Thus, most interpretations rely on clinical features and structural MRI findings, making it difficult to distinguish post-infectious parkinsonism from coexisting or drug-induced movement disorders.

Movement disorders in toxoplasmosis arise from structural injury to basal ganglia, thalamus, and subthalamus, combined with glutamate dysregulation, microvascular obstruction, and dopaminergic disruption. These mechanisms, alone or combined, explain varied presentations. Prompt recognition, focused imaging, and timely anti-*Toxoplasma gondii* therapy are crucial.

## Data Accessibility Statement

All data generated or analyzed in this study are included within the published article and its supplementary materials.

## Additional Files

The additional files for this article can be found as follows:

10.5334/tohm.1093.s1 Supplementary Tables. Supplementary Tables 1–3.

10.5334/tohm.1093.s2 Supplementary Item 1. Evaluation of the methodological quality of case reports and case series.

10.5334/tohm.1093.s3 Supplementary Item 2. PRISMA checklist.
