# Supplementary material for: Movement Disorders in Toxoplasmosis: A Systematic Review
Source: Tremor Other Hyperkinet Mov (N Y). 2025 Sep 30;15:48. doi: 10.5334/tohm.1093 (PMC12493030; doi:10.5334/tohm.1093)
Supplement: Supplementary Tables. — Supplementary Tables 1–3. [file tohm-15-1-1093-s1.pdf]

Supplementary Table 1: Case-wise Clinical, Neuroimaging, Treatment, and Outcome Details of Hyperkinetic Movement Disorders in Central Nervous System Toxoplasmosis (n = 42)

Author/Year	Country	Age/Sex	Immune Status	CD4 count	Duration of illness	Mode of Infection	Diagnostic Method for Toxoplasmosis	CNS Involvement Pattern	Movement Disorder Type	Onset Relative to Infection	Duration of Movement Disorder	Severity of Movement Disorder	Other Neurologic Features	Neuroimaging	Lesion Location and type	Anti-Toxoplasma Treatment	Duration of Treatment	Symptomatic Therapy for movement disorders	Outcome	Follow-up	Pathophysiological Hypothesis
Violante-Villanueva et al., 2023	Mexico	29/M	Untreated HIV	129 cells/µL	41 days	Likely oral (uncooked meat or contaminated food/water)	Positive serum toxoplasma IgG (>150 IU/mL), MRI findings	Left basal ganglia lesion	Right hemichorea-hemiballismus	Initial manifestation	41 days	Severe, large-amplitude violent movements	Dysarthria, abnormal facial movements	A ring-enhancing lesion in the left subthalamic region with surrounding edema; spectrosco	Ring-enhancing lesion in left subthalamic nucleus with perilesional ede	TMP-SMX, pyrimethamine + clindamycin	6 weeks	Haloperidol, aripiprazole, clonazepam, amantadine	Clinical and radiological improvement	Not specified	Toxoplasma abscess involving subthalamic nucleus disrupting basal ganglia circuits

														py reveals peaks of choline and N-acetylcysteine.	ma and meningeal enhancement						leading to hemichorea-hemiballismus
Rocha-Cadman et al., 2024	USA	54/M	Post-hematopoietic stem cell transplant, immunocompromised	Not specified	Subacute (weeks to months)	Reactivation post-HSCT (initially seronegative recipient)	CSF PCR positive for Toxoplasma, MRI findings	Multiple ring-enhancing lesions in thalami, basal ganglia, frontal lobes	Left-sided hemichorea–hemiballismus	Post-transplant	Persistent (≥1 year)	Severe; disabling left upper extremity hemiballismus	Delirium, confusion, hallucinations, weakness, neurocognitive deficits	MRI: multiple ring-enhancing lesions in thalami, basal ganglia, frontal lobes	Bilateral basal ganglia and thalamic ring-enhancing lesions	IV clindamycin, oral pyrimethamine with leucovorin; later sulfadiazine added	≥1 year	Tetrabenazine, Botox injections, olanzapine, quetiapine, haloperidol	Mental status improved, hemichorea–hemiballismus persisted	Long-term follow-up with persistent neurological and psychiatric symptoms	Basal ganglia involvement by Toxoplasma gondii disrupting motor circuits in immunocompromised host
Önder, 20	Turkey	41/F	AIDS	Not spec	9 months	Reactivation	Serology posi	Mesencephalon and	Holmes tremor	9 months	Progressive	Moderate to	Right hemip	MRI: ring-enhancing	Left thalamus,	Trimethoprim-sulfa	≥6 months	Levetiracetam,	Partial improve	Ongoing mul	Delayed secondary

23				ified		of latent toxoplasmosis	tive for HIV and Toxoplasma IgG, MRI findings	thalamus abscesses		after diagnosis	onset, >3 months	severe; tremor and dystonia impairing ADLs	aresis, central facial palsy, visual impairment, breast cancer (later)	abscess with edema, later reduced	internal capsule, mesencephalon; second abscess near right lateral ventricle	methoxazole, azithromycin, dolutegravir, tenofovir/emtricitabine		clonazepam (discontinued), referred for DBS	ment in tremor, persistent dystonia	tidisciplinary follow-up; oncology treatment initiated	degeneration affecting cerebellothalamic and nigrostriatal circuits after toxoplasma abscess
Dimal et al., 2021	Philippines	56/M	HIV-positive (newly diagnosed)	96 cells/μL	3 months	Likely reactivation of latent toxoplasmosis and	Serologic tests (Toxoplasma IgG +); CSF Xpe	Multiple enhancing lesions in basal ganglia, thalami, left frontal	Right-sided hemichorea-hemiballismus	Concurrent with diagnosis of co-infection	Improved over weeks with treatment	Moderate; impaired motor control of right lim	Mild cognitive impairment, no focal weakness	MRI: bilateral basal ganglia and thalamic ring-enhancing lesions	Bilateral basal ganglia and thalamic lesions; mor	Pyrimethamine, sulfadiazine, leucovorin, antitubercular therapy (HRZ	6 weeks intensive phase; ART and ATT continued	Haloperidol, clonazepam	Clinical improvement in movement disorder and general	Not explicitly stated	Lesions in basal ganglia disrupting indirect pathway; dual pathology

						co-infection with tuberculosis	rt MTB/RIF positive; MRI	cortex				bs			e on left	E), ART			condition		(TB and toxoplasmosis) contributing to hyperkinetic movement
Rissardo et al., 2020	Brazil	50/F	Immunocompetent	Not applicable	5 days initial symptoms; tremor 4 months later	Likely ingestion of oocysts (epidemic context)	Serology (IgG, IgM, IgG avidity), CSF PCR for T. gondii	Rhombencephalitis (pons, midbrain), hypertrophic olivary degeneration	Holmes tremor, ocular myoclonus	Initial ataxia and headache; tremor onset 4 months post-treatment	Mild residual symptoms at follow-up	Moderate; managed with symptomatic therapy	Initial: Ataxia, headache; Later: Holmes tremor, ocular myoclonus	1st MRI: Left pontine lesion; 2nd MRI: Hypertrophy of left inferior olivary nucleus	Left pons, inferior olivary nucleus	Pyrimethamine, sulfadiazine, leucovorin	1-month initial treatment; long-term follow-up ongoing	Clonazepam and levodopa	Initial full recovery, later mild improvement after recurrence	Follow-up over several months; no complete resolution	Trans-synaptic degeneration (Guillain–Mollaret triangle) due to pontine lesion causing hypertrophic olivar

																					y degeneration and tremor
Reyes et al., 2018	Trinidad and Tobago	39/M	Immunocompetent	Not applicable	1 week	Likely ingestion of oocysts from cat exposure	Serum and CSF IgG and IgM positive for Toxoplasma; HIV negative; CSF analysis	Mild subclinical encephalitis; normal neuroimaging	Sleep-related multifocal myoclonus	Acute/subacute onset	7 days	Moderate; resolved completely	Febrile illness, flu-like symptoms	Normal MRI, CT, and EEG	No structural lesion identified	Trimethoprim-sulfamethoxazole IV then oral	2 weeks IV then oral maintenance	None given; resolved without symptomatic treatment	Complete resolution within 1 week	Asymptomatic at 1-year follow-up	Subclinical encephalitis due to Toxoplasma-induced GABA inhibition and NMDA activation causing cortical/subcortical myoclonus
Abdel	US	51	HIV-positi	44 ce	Initial	Reacti	MRI findi	Right midb	Holmes	Delaye	Persist	Moder	Right	Ring-enha	Right	Pyrimetha	Standard	Carbido	Persist	12 yea	Holmes

Razek & Venna, 2018	A	/M	ve, ART non-compliant	lls/mm^3	presentation in 2004; tremor developed 4 months later	vation of latent infection	ngs; positive serum Toxoplasma IgG; prior history of CNS toxoplasmosis	rain ring-enhancing lesion with edema extending to right thalamus	tremor (tongue and hand)	d (4 months after lesion)	ent >12 years	ate; partially socially limiting	3rd nerve palsy; left face, arm, leg weakness	ncing lesion in right midbrain extending into thalamus, with edema	midbrain and right thalamus	mine, sulfadiazine, leucovorin	course; tremor persisted long term	pa-levodopa	ent tremor with partial response	rs; no remission or progression	tremor due to lesion in thalamus/midbrain disrupting cerebellar outflow and nigrostriatal pathways
Cambrea et al., 2017	Romania	28/F	HIV-positive, poor adherence to cART	33 cells/mm^3	Not clearly stated (relapse occurred in 2016)	Vertical transmission (infected during infancy,	Presumptive diagnosis: MRI + clinical response to trea	Multiple bilateral cerebral lesions, including basal ganglia, thalamus, midb	Hemiballismus (right limbs)	Occurred after treatment nonadherence and new lesi	Several weeks to months (partial improvement over 5	Moderate to severe; persistent despite initial therap	Initial left-sided motor deficit, later right-sided he	Multiple ring-enhancing lesions with edema; mass effect, T1/T2 imaging	Right caudate nucleus head and left subthalamic nucleus	Cotrimoxazole + Clindamycin	6 weeks	Haloperidol, trihexyphenidyl, valproate (later changed to clon	Improved but persistent symptoms	Follow-up at 5 months with improvement	Relapse likely due to poor adherence; lesion in subthalamic nucleus implic

						Romanian cohort)	tment	rain, frontal lobe		on development	months)	y	miballismus					azepam)			ated in hemiballismus
Reyes et al., 2016	Trinidad and Tobago	22/M	HIV-AIDS (newly diagnosed) + secondary syphilis	3 cells/mL at baseline; 121 cells/mL at 4 months	1 month cognitive decline + 10 days involuntary movements	Likely reactivation (IgG rise, no biopsy)	Serology (IgG rise ×4), MRI	Basal ganglia, thalamus, frontal, right parietal-temporal	Choreoathetosis → dystonia	Subacute	Resolved within 10 days post-treatment	Initially moderate; rapidly reversed	Cognitive decline, MMSE 5/30, improved to 30/30	Hyperintense T2 FLAIR lesions, restricted diffusion, improvement on follow-up MRI	Bilateral basal ganglia, thalamus, right parietal-temporal, frontal lobes	Trimethoprim-sulfamethoxazole + cART (lopinavir-ritonavir, tenofovir-emtricitabine)	Trimethoprim-sulfamethoxazole: 4 months; ART: 4 months	Haloperidol, phenytoin, carbamazepine	Full clinical recovery	MRI, CD4, MMSE follow-up at 12 weeks and 4 months	Lesions in basal ganglia, thalamus, putamen, subthalamus postulated to underlie movement disorders in HIV-AIDS with toxoplasmosis
Berlot et	Sloven	48/	HIV-positive	78	1 day	Unknown	CSF PC	Left parietal	Paroxysmal	Acute	~2 weeks	Moderate	Secondary	MRI: ring-enha	Left parietal	Antiretroviral +	Not specified	Oxcarbazepi	Complete	6 month	Structural lesion

al., 2015	ia	M	(newly diagnosed AIDS)			(undiagnosed HIV)	R for T. gondii	and right occipital ring-enhancing lesions	jerky leg movements (right leg)				GTCS, confusion	ncing lesions with edema in parietal and occipital lobes	and right occipital lobes; abscesses	Toxoplasma therapy (unspecified)		ne	resolution after 2 weeks; asymptomatic at 6 months	s	near leg somatotopy possibly triggered functional jerks; overlap of organic and functional causes; disturbed proprioception and agency
Wu et al., 2014	United Kingdom	47/F	Immunocompromised (AIDS)	20 cells/mm^3	1 month (fever, malaise),	Disseminated toxoplas	Isolation of Toxoplasma gondii	Red nucleus lesion (midbrain)	Holmes’ tremor (rubral tremor)	~8 weeks after hospitalizat	Persistent	Large amplitude, slow	Right hemiparesis, right	MRI: left red nucleus lesion, bilate	T2-enhancing lesion in left	Standard anti-toxoplasma regimen	Not specified	Botulinum toxin (Botox) inje	Improved right-sided stre	Annual MRI, residual glio	Lesion of cerebello-rubro-thalamic

					tremor noted 8 weeks post-admission	mosis (CSF, lymph node, bronchial washings)	in CSF, lymph node, bronchial washings			ion		(3–4 Hz), affecting right upper limb, functionally limiting	facial weakness, dysphasia, increased tone	ral lesions, gliosis and volume loss	red nucleus, consistent with gliosis	(not individually detailed)		ctions, dorsal splint	ngth, tremor partially responsive to Botox	sis and volume loss noted	pathways; involvement of dopaminergic system postulated
Mishra et al., 2014	India	78/M	Immunocompetent	Not applicable	3 days	Stroke (right basal ganglia bleed)	Not applicable	Basal ganglia bleed	Hemiballismus-hemichorea (left side)	Acute	6 days	Severe; interfering with activity	Left extensor plantar, mild left weakness	Multiple small enhancing disseminated lesions of toxoplasmosis	Right basal ganglia hemorrhage	Not applicable	Not applicable	Haloperidol (discontinued due to dystonia), Tetrabenazine, Sodium	Complete recovery	3 months; asymptomatic	Post-stroke basal ganglia lesion causing hemiballismus

																		valproate			
		36/M	HIV positive (also on ATT)	120	4 days	CNS toxoplasmosis	Serum IgM antibodies, MRI, CSF	Basal ganglia, thalamus, right cerebellum	Hemichorea (left side)	Acute	Approx. 2 weeks until improvement	Severe; disabling choreiform movements	Mild left weakness, seizures, hepatitis (due to therapy)	Multiple small enhancing disseminated lesions of toxoplasmosis	Basal ganglia (caudate, pallidum), thalamus, cerebellum	Sulfadiazine + pyrimethamine	At least 1 month (discharged on therapy)	Haloperidol, Valproate, Tetrabenazine	Complete recovery	2 months; asymptomatic, MRI showed resolution	Toxoplasma-induced basal ganglia inflammation
Moccia et al., 2013	Italy	40/M	HIV-positive	67 cells/mm³	Not specified	Likely reactivation	MRI and serology	Right basal ganglia + cerebral cortex	Chorea (left side)	Acute	Few weeks (improved by discharge)	Moderate	No major findings apart from chorea	MRI: enhancing lesions	Right basal ganglia, fronto-parietal cortex	Pyrimethamine + Sulfadiazine	Not specified	Haloperidol	Marked improvement at discharge	Not mentioned	Chorea due to basal ganglia lesions from toxoplasmosis
		41/F	HIV-positive	54 cells/m	Not specified	Likely reactiv	MRI and serolog	Basal ganglia,	Chorea (right side)	Acute	Few weeks	Moderate	Memory com	MRI: ring-enhancing	Basal ganglia,	Pyrimethamine +	Not specified	Haloperidol	Marked improve	Not specified	Basal ganglia disru

				m^3		ation	y	periventricular region					plaints, mood disturbances	lesions with edema	subcortical white matter	Sulfadiazine			ment		ption causing choreiform movements
Rabhi et al., 2011	Morocco	40/M	HIV-positive	34 cells/mm^3	1 month	Likely reactivation	MRI + positive IgG serology for Toxoplasma gondii	Basal ganglia and thalamic involvement	Hemichorea-hemiballismus (left side)	Initial presentation	Improved within weeks	Severe (interfered with activities of daily living)	Mild left hemiparesis	MRI: multiple hypointense lesions with ring enhancement and edema	Right thalamus and basal ganglia	Pyrimethamine, sulfadiazine, folinic acid, corticosteroids	6 weeks	Haloperidol	Marked clinical improvement	Short-term follow-up, symptoms resolved	Lesions affecting motor pathways in subthalamic and thalamic regions likely disrupted inhibitory control, causing hyperkinetic movement

Lekoubou et al., 2010	Cameroon	35/M	HIV-positive (AIDS)	14 cells/mm^3	1 week before hospital admission (fatigue, speech issues); tremor developed on day 8	Not explicitly stated, presumed reactivation in AIDS	CT scan (ring-enhancing lesion), positive IgG antibodies (1/1012)	Right posterior thalamus, internal capsule, edema to midbrain	Holmes tremor (postural, intention, mild rest component)	18 days from initial neurological deficit to tremor onset	8 days	Moderate; resolved with treatment	Cerebellar dysarthria, confusion, hemiparesis, Babinski sign	CT: ring-enhancing lesion in thalamus with edema to upper midbrain	Right thalamus and internal capsule; toxoplasma abscess	Sulfadiazine + Pyrimethamine + Methyl prednisone	At least 3 weeks (duration until discharge)	Clonazepam + Trihexyphenidyl	Complete resolution of tremor; normal speech and motor power on discharge	3 weeks in hospital (no long-term follow-up provided)	Disruption of cerebello-thalamo-cortical and/or dentato-rubro-olivary pathways; possible secondary neuronal degeneration; nigrostriatal involvement not crucial
He	Br	3	HIV-	32	Not	Re	MRI	Basa	Tic	Initi	Ap	Mo	Con	MRI:	Bas	Pyrim	6	Risp	Mar	Not	Basal

nriques Aquino et al., 2010	azil	7/M	positive (AIDS)	cells/mm^3	precisely stated; subacute progression over weeks	activation in advanced HIV	findings + positive Toxoplasma serology (IgG) + clinical response	l ganglia + frontal and parietal lobes	disorder (simple motor tics, vocalizations)	al manifestation	proximately 1 month	derate	fusion, apathy, partial disorientation	multiple hyperintense lesions with ring enhancement	al ganglia, frontal and parietal cortex	ethamine + Sulfadiazine + Folinic acid	weeks	eridone	ked improvement of tics and behavior	mentioned	ganglia and frontal lobe lesions led to dysregulation of cortico-striato-thalamo-cortical circuits involved in tic generation
Midi et al., 2008	Turkey	37/M	HIV-positive (AIDS)	<50 cells/mL	~1 month	Likely blood transfusion	Brain biopsy, anti-Toxo IgG positive,	Multiple cerebral and cerebellar abscesses	Generalized choreoathetosis	~1 month after initial symptoms	<1 week (until death)	Severe	Altered consciousness, disorientation, bilat	MRI: Multiple enhancing cerebral and cerebellar lesion	Bilateral cerebral and cerebellar abs	Pyrimethamine	Short (death within a week)	Antidopaminergic drugs (ineffective)	Death due to bacterial respiratory infectio	Until death	Bilateral subcortical involvement due to toxoplasm

							MRI						eral papilledema, hyperreflexia, clonus	s	cesses				n		a abscesses in thalamus, caudate, putamen, globus pallidus, midbrain, and internal capsule caused generalized chorea
Zuniga et al., 2005	Argentina	34/F	HIV-positive	Not reported	Since 1998, dystonia onset 4 month	Presumed sexually transmitted	MRI with basal ganglia lesions + positive T.	Bilateral basal ganglia lesions	Segmental dystonia (left upper limb), neck dystonia	Delayed onset (4 months after encephalitis	Persistent (multiple years)	Severe, disabling	Mild neck dystonia, history of encephalopathy	Initial MRI: ring-enhancing lesions in basal ganglia; follow-	Bilateral basal ganglia granulomas	Pyrimethamine and sulfadiazine	Not specified; effective in radiological resolution	Botulinum toxin injections every 3 months	Marked improvement with botulinum toxin desp	At least 3 years (2002–2005)	Basal ganglia lesions (caudate, thalamus, lentiform

					s post-treatment		gondii serology			)			, seizures	up: resolution					ite persistent dystonia		nucleus) disrupt thalamocortical and striatal circuits; possible sequelae of necrosis
Factor et al., 2003	USA	36/M	HIV-positive	14 cells/μL	1 month of altered mental status before dystonia	Not specified (HIV-related)	CT scan showing right basal ganglia lesion, positive serology	Right basal ganglia abscess	Hemidystonia (left-sided)	During hospitalization for cerebral toxoplasmosis	Several months	Moderate to severe	Left hemiparesis, cognitive impairment	CT: Enhancing lesion in right basal ganglia	Right basal ganglia toxoplasmic abscess	Sulfadiazine, pyrimethamine, folinic acid	Not specified	Baclofen and clonazepam	Modest improvement with symptomatic medications	Several months	Basal ganglia disruption by abscess impaired sensorimotor circuits, causing dystonia
Pezzini et	Italy	38/M	HIV-positive	Not men	Subacute ons	Likely reactiv	CT and MRI findi	Midbrain abscess	Holmes tremor	Developed afte	Persistent unti	Severe dis	Mild hemipare	MRI: ring-enhancing	Midbrain lesi	Pyrimethamine +	6 weeks	Levodopa + Clo	Marked reducti	Several month	Damage to red nucle

al., 2002				tioned	et over weeks	ation in HIV	ngs + serology	due to toxoplasmosis		r abscess formation	l symptomatic treatment	abling tremor	sis, vertical gaze palsy	lesion in midbrain	on with mass effect and contrast enhancement	Sulfadiazine + Folinic acid		nazepam + Propranolol	on in tremor	s follow-up	us and surrounding midbrain structures causing combined rest, action, and postural tremor (Holmes tremor)
Piccolo et al., 1999	Italy	27/M	HIV positive, AIDS	Not mentioned	1 year post AIDS diagnosis	IV drug use	Serology, MRI	Left subthalamic nucleus	Right hemichorea-hemiballismus	2 years post-HIV	2 months	Moderate	None	Ring-enhancing lesion on MRI	Left subthalamic nucleus; Toxoplasma abscess	Pyrimethamine, sulfamethopyrazine	Not specified	Not mentioned	Complete resolution	1-month post-treatment	Damage to subthalamic nucleus leading to disinhibition

																					of thalamocortical circuits
		56/M	HIV positive, AIDS	21	1 year	Heterosexual transmission	PCR for JC virus in CSF (PML)	Right internal capsule, bilateral periventricular white matter	Left hemifacial and limb chorea	Post-AIDS diagnosis	Persistent till death	Severe	Ataxia, hypotrophy, reduced reflexes	MRI hyperintense lesions, diffuse atrophy	Right pons, internal capsule (PML)	Not applicable	Not applicable	Not mentioned	Death due to AIDS complications	None	Involvement of basal ganglia efferent pathways
		43/M	HIV positive, AIDS	50	Subacute onset	Bisexual contact	HIV antigen in CSF; MRI (no toxoplasma)	Bilateral white matter	Generalized chorea	6 years post-HIV	Resolved in few months	Mild	Mild cognitive decline	MRI hyperintensities	White matter, internal capsules	None	Not applicable	Not mentioned	Improved with AZT	5 months	HIV encephalopathy affecting subcortical structures
		35/M	HIV positive, AIDS	10	6 years post-HIV	IV drug use	Autopsy (Toxoplasma	Bilateral basal ganglia and thala	Generalized chorea	Subacute	Till death	Severe	Left hemiparesis, febrile	Multiple abscesses, MRI lesions	Basal ganglia, thalamus;	Empirical	Not specified	Not mentioned	Death (pneumonia)	Autopsy performed	Extensive caudate involvement

							tachyzoites)	mus							Toxoplasma abscess						
		32/M	HIV positive, AIDS	Not mentioned	1 year post AIDS	IV drug use	Not applicable	Diffuse edema	Buccofaciolingual dyskinesia	During septic shock	Brief	Moderate	Sepsis, coma	CT: cerebral edema	Diffuse; bacterial meningitis	None	Not applicable	Not mentioned	Death	No autopsy	Catecholamine-induced (iatrogenic)
Micheli et al., 1997	Argentina	39/M	HIV-positive	160/μL	~2 months (symptoms)	IV drug use, alcoholism	Elevated serum IgG (1:2048), MRI/CT	Thalamus + internal capsule	Postural and action tremor	After hemiparesis recovery	~11 months	Mild residual at 11 months	Initial right hemiparesis, asthenia, visual distance judgment issues	MRI: hyperintense T2 lesion in left posterior thalamus + posterior internal capsule; resolved on follow-up	Left posterior thalamic toxoplasma abscess	Pyrimethamine, sulfadiazine, zidovudine	Not stated	Diazepam (IV) administered	Mild residual tremor after 11 months	11 months follow-up	Thalamic lesion disrupted cerebellar/brainstem-thalamic inputs; tremor due to residual structural damage post-

																					abscess resolution; analogous to thalamic stroke tremor cases
Maher et al., 1997	Canada	40/M	HIV+ (AIDS dementia complex)	30/mm³	6 months (before onset of myoclonus)	Not specified	No toxoplasmosis	Dementia, brainstem involvement (autopsy)	Generalized myoclonus (startle-like)	Terminal stage of AIDS	Persisted until death (~2 weeks after evaluation)	Persistent, moderate	Severe dementia, parkinsonian gait, dysarthria	CT: generalized atrophy	Brainstem nuclei (autopsy: nucleus reticularis)	Not given	N/A	Not given	Died within 2 weeks	Short, until death	Subcortical myoclonus; brainstem involvement
Maggi et al., 1996	Italy	27/M	AIDS	50/mm³	Not specified	IV drug user	Antibodies, CT/MRI, respon	Left Hemichoreoathetosis	Hemichoreoathetosis	At presentation	30 days	Complete	Confusion, right hemiparesis	MRI: multiple granulomatous lesions in	Left putamen, frontal, peritrig	Pyrimethamine + sulfadiazine	≥3 months	Not specified	Complete recovery	No follow-up imaging	Subthalamic nucleus and pallidal/tha

							se to therapy						sis, aphasia	basal ganglia, temporal, occipital, subthalamic, and mesencephalic regions with edema; marked reduction on follow-up.	onal necrotic lesions with edema; additional in thalamus and insula						lamic pathway involvement
		31/M	AIDS	30/mm³	Not specified	Homosexual	Antibodies, CT/MRI, response to ther	Left hemichoreoballismus	Choreoballism	At presentation	30 days	Marked	None specified	MRI: multiple granulomatous lesions in basal ganglia,	Right caudate, right subthalamic, nec	Pyrimethamine + sulfadiazine	≥3 months	Not specified	Marked improvement	4 months	Subthalamic/pallidal and pallidal/thalamic involvement

							apy							temporal, occipital, subthalamic, and mesencephalic regions with edema; marked reduction on follow-up.	rosis in right pallidus						
		32/M	AIDS	30/mm³	Not specified	IV drug user	Antibodies, CT/MRI, response to therapy	Left hemichoreoathetosis	Hemichoreoathetosis	At presentation	10 days	Complete	Confusion	MRI: multiple granulomatous lesions in basal ganglia, temporal, occipi	Right subthalamic, mesencephalic, occipital, bilateral	Pyrimethamine + sulfadiazine	≥3 months	Not specified	Recovery	3 months	Subthalamic/pallidal and pallidal/thalamic involvement

														tal, subthalamic, and mesencephalic regions with edema; marked reduction on follow-up.	temporal granulomatous lesions						
Garretto et al., 1995	Argentina	26/M	HIV positive (AIDS)	218/mm3	Approximately 2 months until hemichorea onset	Not specified, likely sexual	Positive serology (IFA 1:1024, HA 1:4096), MRI	Multiple lesions including right frontal, left temporo-occipital and later right	Hemichorea (left-sided)	12th day of treatment	Persistent after 6 weeks of treatment	Moderate, impaired gait	Initial left hemiparesis, seizures	MRI: Right frontal and left temporo-occipital lesion initially; later right peduncular	Right frontal, left temporo-occipital, and right cerebral	Pyrimethamine, sulfadiazine, folinic acid	6 weeks	Haloperidol (ineffective)	Motor deficit improved, hemichorea persisted	Imaging confirmed lesion reduction, no resolution of he	Lesion in contralateral cerebral peduncle likely cause; HIV and Toxoplasma-

								peduncle						lesion	peduncle; contrast enhancing with edema					michorea	induced basal ganglia dysfunction may contribute
Tedrus et al., 1994	Brazil	54/M	HIV Positive	Not stated	7 days (onset of symptoms)	Not known	CT scan, positive IgG for Toxoplasma in CSF, positive ELISA for HIV	Thalamus and subthalamus	Hemiballismus	Initial manifestation	Progressive over time	Severe	Hepatomegaly; no other CNS signs noted	CT: hypodense lesion with mass effect and contrast enhancement in left thalamus/subthalamus	Left thalamus and subthalamus with edema	Sulfadiazine and pyrimethamine started after haloperidol failure	Not stated	Haloperidol (15 mg/day)	No significant improvement	Not stated	Toxoplasmosis-induced lesion in the thalamus/subthalamus disrupting extrapyramidal circuits
Micheli et al., 1994	Italy	11/F	Immunocompetent	Not reported	4 weeks before hospital	Not explicitly stated; acq	Serum antibody titres	No neuroimaging abnormalities;	Hemidystonia (right side of body)	Subacute onset, weeks	Approximately 2 months	Severe; interfering with writi	Athetosis, ballistic-like mov	CT and MRI normal	No visible lesions; presume	Pyrimethamine, spiramycin, and sulfon	1 year	Trihexyphenidyl, delorazepam	Complete recovery within 2 mon	6 years	Focal cerebral vasculitis or encephalitis

					ization to full recovery in ~2 months	uired toxoplasmosis	(IgG-IFA, Dye test, IgM-ISA); CSF negative	suspected cerebral vasculitis or focal encephalitis		after possible infection		ng and object handling	ements, no cranial nerve or sensory deficits		d lenticulostriatal vascular involvement	amides		(ineffective)	ths; EEG normalized in 8 months		affecting lenticulostriatal system
Nath et al., 1993	Canada	33/M	Immunocompromised (AIDS)	<50 cells/mm^3	Several weeks	Reactivation of latent infection	Clinical, neuroimaging, confirmed at autopsy	Bilateral basal ganglia, thalamic, cortical	Generalized chorea, dystonia, parkinsonism	Subacute onset with evolving symptoms	~4 weeks until death	Severe	Dementia, behavioral changes, bradykinesia	MRI	Basal ganglia, thalamus, cortical (multiple ring-enhancing lesions)	Pyrimethamine, sulfadiazine, leucovorin	<4 weeks	Not specified	Death	Autopsy findings confirmed diagnosis	Toxoplasma-induced lesions in basal ganglia and thalamus disrupting motor circuits
de Mattos et al.,	Brazil	31/M	HIV-positive (AIDS)	Not reported	At presentation	Presumed opport	CT imaging, clinical	Bilateral frontal lobes with	Hemichorea-hemiballismus	Initial manifestation	4 months (till dea	Persistent despite tre	Intracranial hyperte	CT brain	Frontal ring-enhanci	Sulfadiazine and pyrimetha	Not stated	Neuroleptics (inefecti	Death from respirato	Until death (4 mo	Toxoplasma abscess in basal

1993						unistic	response to therapy	ring-enhancing lesions			th)	atment	nsion		ng lesions, improved after treatment	mine		ve)	ry failure and sepsis	nths)	ganglia region possibly affecting motor circuits
		35/M	HIV-positive (AIDS)	Not reported	2 years after AIDS diagnosis	Presumed opportunistic	CT scan showed basal ganglia lesion	Right basal ganglia	Hemichorea-hemiballismus (including facial involvement)	2 years post-AIDS diagnosis	≥2 years (improved after CNS lymphoma therapy)	Severe	Later developed CNS lymphoma	CT scan	Initial low-density lesion in basal ganglia, later mass lesions in frontal, parietal, occipital lobe	Pyrimethamine and sulfadiazine	Not stated	Thioridazine (effective)	Improved after radiotherapy for lymphoma	≥2 years	Basal ganglia toxoplasmosis and later lymphoma affecting motor circuits

															s						
Tolge & Factor / 1991	USA	34 / M	Immunocompromised (AIDS)	Not Reported	Few weeks	Reactivation	Clinical + Neuroimaging	Basal ganglia	Focal dystonia (left hand)	Subacute onset	Persistent during hospitalization	Moderate	Right hemiparesis, mild cognitive changes	CT: ring-enhancing lesion	Right basal ganglia	Pyrimethamine + Sulfadiazine + Folinic acid	4 weeks	Not clearly mentioned	Improvement	Not reported	Lesion-induced disruption of motor circuits (putamen)
Koppel & Daras, 1990	USA	35 / M	Presumed AIDS (former IV drug user, oral candidiasis, febrile, cachectic)	Not reported	Unknown prior to admission	Presumed opportunistic infection in AIDS	CSF Toxoplasma IgG positive (1:16); CT response to therapy	Midbrain ring-enhancing lesion crossing midline	Rubral tremor (left arm, leg, jaw)	Presenting symptom	Persistent until death (80 days)	Severe; interfered with voluntary control	Right hemiplegia, left 3rd nerve palsy, dysarthria, altered mental status	CT brain	Midbrain (left > right); ring-enhancing abscess	Pyrimethamine, trisulfapyrimidine, folinic acid, dexamethasone	4 weeks	Trihexyphenidyl (partial), clonazepam, primidone, levodopa (ineffective); isoniazid improve	No improvement in tremor; death due to cardiorespiratory arrest	80 days (until death)	Lesion in midbrain (dentatothalamic and dentato-olivary system) caused rubral tremor

																		d tremor			
Sanchez-Ramos et al., 1989	USA	33/F	HIV-positive (AIDS)	Not reported	2 weeks before presentation	Not reported; presumed HIV/AIDS	Positive Toxoplasma serology; CT findings; response to anti-Toxoplasma therapy	Multiple enhancing cerebral lesions with edema	Hemichorea-hemiballismus (left upper > lower limb)	Initial presentation	~20 days during hospitalization; improved with treatment	Severe; required assistance for walking; frequent falls	Headache, double vision, vomiting, light headedness	CT scan brain	Right subthalamic nucleus, right thalamus, right cerebellum, left caudate, left frontal WM	Pyrimethamine, sulfadiazine, clindamycin, dexamethasone	~3 weeks during first admission; second course 2 months later	Possibly dexamethasone; no specific antidyskinetic mentioned	Improved with treatment; relapse without movement disorder; died of meningitis	~5.5 months until death	Subthalamic nucleus lesion with edema hypothesized to cause hemichorea-hemiballismus
Carrazana et al., 1989	USA	54/M	HIV-positive (AIDS, confirmed by ELIS	Not reported	2 weeks before admission	IV drug use	CT scan + postmortem pathology	Left subthalamic nucleus abscess with	Unilateral akathisia (right-sided)	Initial presenting symptom	~4 weeks until death	Severe; disabling restlessness	Anxiety, restlessness, hyperreflexi	CT: enhancing lesion in left subthalamic regio	Left subthalamic nucleus + parietal	Pyrimethamine and sulfadiazine	~2 weeks (stopped due to rena	Haloperidol worsened akathisia	Progressive renal failure; death	1 month after onset	Subthalamic nucleus lesion led to basal gangl

			A and Western blot)				confirmation of Toxoplasma cysts	surrounding edema				ss, worsened with haloperidol	a, right Babinski, gait disturbance	n with edema and shift	lobe necrotic lesions (Toxoplasma)		l failure)				ia edema affecting extrapyramidal circuits; first report of unilateral akathisia in AIDS

ADLs – Activities of Daily Living; AIDS – Acquired Immune Deficiency Syndrome; ART – Antiretroviral Therapy; ATT – Antitubercular Therapy; AZT – Zidovudine (Azidothymidine); cART – Combination Antiretroviral Therapy; CD4 – Cluster of Differentiation 4; CNS – Central Nervous System; CSF – Cerebrospinal Fluid; CT – Computed Tomography; DBS – Deep Brain Stimulation; ELISA – Enzyme-Linked Immunosorbent Assay; FLAIR – Fluid-Attenuated Inversion Recovery; GTCS – Generalized Tonic-Clonic Seizure; HA – Hemagglutination Assay; HIV – Human Immunodeficiency Virus; HRZE – Isoniazid, Rifampicin, Pyrazinamide, Ethambutol; HSCT – Hematopoietic Stem Cell Transplant; IFA – Immunofluorescent Antibody; IgG – Immunoglobulin G; IgM – Immunoglobulin M; IV – Intravenous; MMSE – Mini-Mental State Examination; MRI – Magnetic Resonance Imaging; NMDA – N-Methyl-D-Aspartate; PCR – Polymerase Chain Reaction; PML – Progressive Multifocal Leukoencephalopathy; TMP-SMX – Trimethoprim-Sulfamethoxazole; WM – White Matter

References

1. Violante-Villanueva A, López-Hernández JC, Salas-Alvarado L, García X. 29-year-old male with hemichorea-hemiballismus as the initial manifestation of cerebral toxoplasmosis in the setting of untreated HIV. Arch Neurocien (Mex). 2023;28(3):40-3. doi:10.31157/an.v28i3.429.

2. Rocha-Cadman X, Revilla AR, Cadman K, Root JC. Hemichorea–hemiballismus associated with a case of cerebral toxoplasmosis in a hematopoietic stem cell transplant recipient. Palliat Support Care. 2024;22(3):623–5. doi:10.1017/S1478951524000105.

3. Önder Ö. Holmes tremor in a case of AIDS with toxoplasma abscess: Is a common result of different pathogenetic mechanisms? *J Gazi Univ Health Sci Inst.* 2023;6(1):29–35. doi:10.59124/guhes.1348648.

4. Dimal NPM, Santos NJC, Reyes NGD, Astejada M, Jamora RDG. Hemichorea-hemiballismus as a presentation of cerebritis from intracranial toxoplasmosis and tuberculosis. Tremor Other Hyperkinet Mov (N Y). 2021;11:2. doi:10.7916/d8-jnzs-5f49.

5. Rissardo JP, Caprara ALF, Silveira JOF. Rhombencephalitis secondary to *Toxoplasma gondii* infection: Case report and literature review. *Acta Med Int.* 2020;7(1):51–4. doi:10.4103/ami.ami_78_19.

6. Reyes AJ, Ramcharan K, Giddings SL, Aboh S, Rampersad F. Myoclonic jerks, exposure to many cats, and neurotoxoplasmosis in an immunocompetent male. Tremor Other Hyperkinet Mov (N Y). 2018;8:511. doi:10.7916/D8B86GQC.

7. AbdelRazek MA, Venna N. Unilateral tongue and hand tremor secondary to a brainstem lesion. Mov Disord Clin Pract. 2018;5(3):348. doi:10.1002/mdc3.12617.

8. Cambrea SC, Pascu C, Rugină S. Hemiballismus in a patient with acquired immunodeficiency syndrome and *Toxoplasma* infection. *Rom J Infect Dis.* 2017;20(1):20–5. doi:10.37897/RJID.2017.1.3.

9. Reyes AJ, Ramcharan K, Aboh S, Duke N. Reversible movement disorders due to toxoplasmosis as initial manifestation of HIV-AIDS, with sequential MR and video imaging. BMJ Case Rep. 2016;2016:bcr2016215676. doi:10.1136/bcr-2016-215676.

10. Berlot R, Kramberger M, Dreo J, Pirtošek Z, Kojović M. Blurred boundaries between organic and functional etiology: A man with a jerky leg. J Neurol Sci. 2015;354(1–2):122–3. doi:10.1016/j.jns.2015.04.046.

11. Wu K, Siddiqui A, Andrews T, Holmes P. Holmes' tremor from a lesion of the red nucleus in association with cerebral toxoplasmosis. Basal Ganglia. 2014;4(2):81–3. doi:10.1016/j.baga.2013.12.001.

12. Mishra V, Jain S, Sorabjee J. Combination therapy in hemiballismus-hemichorea syndromes – A report of two cases. Bangladesh J Med Sci. 2014;13(4):484–7. doi:10.3329/bjms.v13i4.17536.

13. Moccia M, Picillo M, Carotenuto A, Barbato F, Gentile I, Orefice G. Movement disorders as presenting symptoms of AIDS. Basal Ganglia. 2013;3(3):175–8. doi:10.1016/j.baga.2013.03.004.

14. Rabhi S, Amrani K, Maaroufi M, Khammar Z, Khibri H, Ouazzani M, et al. Hemichorea-hemiballismus as an initial manifestation in a Moroccan patient with acquired immunodeficiency syndrome and toxoplasma infection: a case report and review of the literature. Pan Afr Med J. 2011;10:9.

15. Lekoubou A, Njouoguep R, Kuate C, Kengne AP. Cerebral toxoplasmosis in Acquired Immunodeficiency Syndrome (AIDS) patients also provides unifying pathophysiologic hypotheses for Holmes tremor. *BMC Neurol.* 2010;10:37. doi:10.1186/1471-2377-10-37.

16. Henriques Aquino CC, Felício AC, Godeiro-Junior C, Santos-Neto D, Pedroso J, Bulle Oliveira AS, et al. Tic Disorder: an unusual presentation of neurotoxoplasmosis in a patient with AIDS. *Case Rep Neurol.* 2010;2(3):145–9. doi:10.1159/000322185.

17. Midi I, Çalişkan HG, Toktaş Z, Sav A, Günal DI. Generalized choreoathetosis in an acquired immune deficiency syndrome patient with cerebral toxoplasmosis. *Turk J Med Sci.* 2008;38(4):377–80.

18. Zuniga C, Lester J, Scorticati MC, Micheli F. Persistent limb dystonia in a HIV positive patient with cerebral toxoplasmosis. J Neurol Sci Turk. 2005;22(4):414–6.

19. Factor SA, Troche-Panetto M, Weaver SA. Dystonia in AIDS: report of four cases. Mov Disord. 2003;18(12):1492–8. doi:10.1002/mds.10602.

20. Pezzini A, Zavarise P, Palvarini L, Viale P, Oladeji O, Padovani A. Holmes' tremor following midbrain Toxoplasma abscess: clinical features and treatment of a case. Parkinsonism Relat Disord. 2002;8(3):177–80. doi:10.1016/s1353-8020(01)00013-x.

21. Piccolo I, Causarano R, Sterzi R, Sberna M, Oreste PL, Moioli C, et al. Chorea in patients with AIDS. Acta Neurol Scand. 1999;100(5):332–6. doi:10.1111/j.1600-0404.1999.tb00406.x.

22. Micheli F, Grañana N, Scorticati MAC, Giannaula RJ, Reboredo G. Unilateral postural and action tremor resulting from thalamic toxoplasmosis in a patient with acquired immunodeficiency syndrome. Mov Disord. 1997;12(6):1044–6. doi:10.1002/mds.870120647.

23. Maher J, Choudhri R, Halliday W, Power C, Nath A. AIDS dementia complex with generalized myoclonus. *Mov Disord*. 1997;12(4):593–7. doi:10.1002/mds.870120421.

24. Maggi P, de Mari M, De Blasi R, Armenise S, Romanelli C, Andreula C, et al. Choreoathetosis in acquired immune deficiency syndrome patients with cerebral toxoplasmosis. Mov Disord. 1996;11(4):434–6. doi:10.1002/mds.870110414.

25. Garretto N, Duarte J, Lucchini L, Motta M, Fernandez Pardal M. Hemichorea in a patient with toxoplasmosis and acquired immune deficiency syndrome. Mov Disord. 1995;10(6):783–784. doi:10.1002/mds.870100624.

26. Tedrus GM, Albertin MC, Fonseca LC. Hemiballismus as initial manifestation of acquired immunodeficiency syndrome: a case report. Arq Neuropsiquiatr. 1994;52(2):263–5. doi:10.1590/S0004-282X1994000200019.

27. Micheli R, Perini A, Duse M. Hemidystonia secondary to acquired toxoplasmosis in a non-immunodeficient patient. Eur J Pediatr. 1994;153(10):731–733. doi:10.1007/BF01954489.

28. Nath A, Hobson DE, Russell A. Movement disorders with cerebral toxoplasmosis and AIDS. *Mov Disord.* 1993;8(1):107–112. doi:10.1002/mds.870080119.

29. de Mattos JP, Rosso ALZ, Corrêa RB, Novis S. Involuntary movements and AIDS: report of seven cases and review of the literature. Arq Neuropsiquiatr. 1993;51(4):491-497. doi:10.1590/S0004-282X1993000400009.

30. Tolge CF, Factor SA. Focal dystonia secondary to cerebral toxoplasmosis in a patient with acquired immune deficiency syndrome. Neurology. 1991; 41(10):1683–1684. DOI: 10.1212/WNL.41.10.1683.

31. Koppel BS, Daras M. “Rubral” tremor due to midbrain toxoplasma abscess. Mov Disord. 1990;5(3):254–256. doi:10.1002/mds.870050313.

32. Sanchez-Ramos JR, Factor SA, Weiner WJ, Marquez J. Hemichorea-hemiballismus associated with acquired immune deficiency syndrome and cerebral toxoplasmosis. Mov Disord. 1989;4(3):266–273. doi:10.1002/mds.870040313.

33. Carrazana E, Rossitch E Jr, Martínez J. Unilateral “akathisia” in a patient with AIDS and a toxoplasmosis subthalamic abscess. Neurology. 1989;39(3):449–450. doi:10.1212/WNL.39.3.449.

Supplementary Table 2: Case-wise Clinical, Neuroimaging, Treatment, and Outcome Details of Hypokinetic Movement Disorders in Central Nervous System Toxoplasmosis (n = 9)

Author/ Year	Country	Age/ Sex	Immune Status	CD4 count	Duration of illness	Mode of Infection	Diagnostic Method for Toxoplasmosis	CNS Involvement Pattern	Movement Disorder Type	Onset Relative to Infection	Duration of Movement Disorder	Severity of Movement Disorder	Other Neurological Features	Neuroimaging	Lesion Location and type	Anti-Toxoplasma Treatment	Duration of Treatment	Symptomatic Therapy for movement disorders	Outcome	Follow-up	Pathophysiological Hypothesis
Mohammadzadeh et al., 2023	USA	64/F	HIV-positive (well-controlled)	216 cells/mm^3	26 years (from initial encephalitis in 1997 to report in 2023)	Reactivation of latent toxoplasmosis	MRI: encephalomalacia, gliosis; history of treated cerebral toxoplasmosis	Right midbrain and bilateral putaminal lesions	Asymmetric parkinsonism with tremor and bradykinesia	Delayed onset (after recovery from toxoplasmosis)	Progressive over decades	Moderate to severe; tremor, bradykinesia, gait instability	Cognitive impairment, imbalance, impaired dexterity	FLAIR and T1/T2* MRI: gliosis, encephalomalacia; DAT scan: symmetric reduced putaminal activity	Right midbrain and bilateral putamina (encephalomalacia and gliosis)	Not reported in the case (toxoplasmosis is treated in 1997)	Not applicable in current report	Levodopa (ineffective), skin biopsy for alpha-synuclein negative	Persistent parkinsonism, no benefit from dopaminergic therapy	Long-term; symptoms stable but progressive	Post-infectious basal ganglia and midbrain damage causing secondary parkinsonism (non-synucleinopathic)
Malaquias	UK	42	HIV-positi	390 cell	Acute	Likely	Positive	Right	Left-sided	Subacut	~1.5	Moder	Mild cog	MRI: right	Right thala	Pyrimethami	6 wee	Trial of	Persist	1.5 yea	Post-infecti

et al., 2023		/M	ve (on ART since 2016)	s/µL	onset in 2021, chronic symptoms for ~1.5 years	reactivation of latent toxoplasmosis	toxoplasma serology; MRI; DaTSCAN	thalamus and posterior putamen lesion	hemiparkinsonism (bradykinesia, rigidity, reduced arm swing)	e after toxoplasmosis diagnosis	years (at time of report)	ate; affecting daily function	nitive impairment (MoCA = 25/30), persistent headache	thalamic and posterior putamen lesion with encephalomalacia; DaTSCAN: reduced uptake in right striatum	mus and posterior putamen encephalomalacia	ne, sulfadiazine, folinic acid, ART	ks	levodopa ineffective	ent parkinsonian symptoms	rs follow-up with stable symptoms	ous degeneration of nigrostriatal dopaminergic pathway causing presynaptic parkinsonism
Donlon et al., 2023	Ireland	59/F	HIV-positive	174 cells/mm^3	18 months (post initial diagnosis of cerebral toxoplasmosis)	Reactivation in setting of HIV	Clinical history, prior antimicrobial therapy, MRI findings	Multiple cerebral ring-enhancing lesions	Right-sided hemiparkinsonism + Left-sided hemidystonia/chorea	Began after initial toxoplasmosis diagnosis	≥18 months	Moderate; bilateral movement disorder with functional impair	None specifically noted beyond movement symptoms	MRI: multiple ring-enhancing lesions in right thalamus, left lentiform nucleus, right	Right thalamus (left hyperkinesia); left lentiform nucleus (right parkinsonism)	Completed antimicrobial therapy for toxoplasmosis	Not specified	Brief risperidone trial not tolerated (orobuccal dyskinesia, mood changes); no further	Persistent symptoms; no improvement reported	Follow-up duration unclear; no further intervention accept	Strategic lesioning of basal ganglia circuits: thalamus affecting motor inhibition (hype

												ment		frontal and temporal lobes				therapy pursued		ed	rkinetic) and lentiform nucleus affecting dopaminergic pathways (hypokinetic)
Malhotra et al., 2017	USA	NA/M	Immunocompetent (HIV-negative)	Not available	3 months	Likely hematogenous dissemination	Autopsy: histopathology and immunohistochemistry positive for T. gondii	Multifocal: frontal lobes, basal ganglia, thalamus, corpus callosum, occipital lob	Ataxia, akinetic mutism, hemiparesis suggests extrapyramidal involvement)	Subacute	Not stated	Severe, progressive	Headache, confusion, memory loss, urinary incontinence, akinetic mutism, flaccid paralysis	MRI: ring-enhancing lesions, edema, mass effect, midline shift	Right basal ganglia, thalamus, corpus callosum, frontal and occipital lobes, brain stem	Not administered (diagnosis made post-mortem)	Not applicable	Not applicable	Death	Post-mortem diagnosis	Necrotizing toxoplasmic encephalitis mimicking glioma; immunocompetent status may delay clinical suspicion

								es, brainstem													
Akinci et al., 2017	Turkey	54/M	HIV-positive, AIDS stage	52 cells/mm³ (baseline), 225 (post-treatment)	6+ months progressive symptoms before diagnosis	Likely reactivation (positive anti-Toxoplasma IgG)	MRI lesion + IgG seropositivity	Left lentiform nucleus + bilateral white matter	Parkinsonism (bradykinesia, postural instability), dementia	Subacute onset, progressive worsening	At least 4–6 months	Moderate to severe initially, dramatic improvement post-treatment	Cognitive decline, aggression, apathy, hallucinations	Hyperintense lesion in left lentiform nucleus, diffuse white matter changes, atrophy	Left lentiform nucleus + bilateral subcortical white matter	Trimethoprim-sulfamethoxazole + cART (lopinavir/ritonavir, tenofovir/emtricitabine)	At least 4 months documented	None specific; movement disorder improved with antiparasitic + antiretroviral therapy	Marked clinical recovery (SMMT from 14 to 29/30)	Follow-up at 1 and 4 months	Basal ganglia toxoplasmosis + HAND (HIV-associated neurocognitive disorder) synergistically producing parkinsonism and dementia
Arbune et al., 2016	Romania	58/F	HIV-positive (Stage C3 AIDS)	7/mm³ at presentation; increased	Sudden coma; progressive recovery over	Not specified (presumed reactivation)	Positive PCR for T. gondii in CSF and HIV serology	Supra- and infratentorial lesions	Parkinsonian syndrome (bradykinesia, tremor,	Following recovery from coma	Several months	Moderate to severe	Tetraparesis, diplopia, depression, choreoat	CT and MRI showing basal ganglia and front	Basal ganglia, subcortical frontal; supra- and	Pyrimethamine, clindamycin, TMP-SMX, plus HAART	Antiparasitic for several weeks; HA	Levodopa, clonazepam, sertraline	Partial recovery; persistent parkins	Improvement over 6–12 months	Direct toxoplasma damage to basal ganglia and dopamine

				to 224/mm^3	6–12 months			including basal ganglia and frontoparietal subcortical areas	rigidity, postural instability)				hetotic movements	oparietal lesions (including ‘bulls eye’ lesions)	infratentorial		ART continued		onism and tremor		dysregulation; HIV effects; possible immune reconstitution syndrome
Murakami et al., 2000	Japan	49/M	HIV-positive (AIDS)	150/μL at admission	3 months prior to diagnosis	Not explicitly mentioned	MRI + Toxoplasma IgG serology	Basal ganglia and parietal subcortex	Bilateral parkinsonism	Initial manifestation of AIDS	At least 6 months	Moderate; disabling initially	Gaze palsy, hyperreflexia, confusion	MRI: ring-enhancing lesions in basal ganglia and subcortex	Right lenticular nucleus + parietal white matter lesions	Clindamycin + pyrimethamine	At least 6 months	Levodopa/carbidopa 300 mg/day	Partial clinical and radiological improvement	6 months	Basal ganglia lesions damaging dopaminergic pathways; low CSF HVA; possible HIV contribution
Maggi et al., 2000	Italy	31/M	HIV-positive (AID	Not specified	HIV diagnosed	Intravenous	MRI + CSF PCR for	Bilateral lent	Akinetic-rigid parkin	Several years	Persistent unt	Severe; disablin	Behavioral abn	MRI: inflammatory	Bilateral lentiform	Pyrimethamine + sulfadi	60 days	Not specified	Deteriorated	July–Dece	Bilateral basal gangli

			S)		in 1991; parkinsonism onset in 1995	drug use	Toxoplasma gondii	iform nucleus and cortical-subcortical involvement	sonism	post-HIV; developed after bilateral involvement	il death (~5–6 months)	g features including gait freezing	ormalities, cranial nerve palsy, coma	and abscess lesions in lentiform nuclei and cortex	nuclei, frontobasal abscess, left cortical-subcortical lesions	azine			neurologically; died at home	mber 1995	a involvement impaired indirect pathway; possible HIV glutamate toxicity
Carrazana et al., 1989	USA	66/F	HIV-positive (AIDS, confirmed by ELISA & Western blot)	Not reported	~4 months prior to death	Presumed blood transfusion	High IgG toxoplasma titers in serum (1:4096) and CSF (1:64), stereotactic biopsy confirmed toxoplasma cysts	Bilateral basal ganglia lesions, anterior limbs of internal capsules	Parkinsonism (rigidity, tremor, bradykinesia)	Subacute onset; ~2 weeks before admission	Persisted until death (4 months total)	Severe; progressed to quadriparesis, contractures	Confusion, fever, lethargy, spasticity, anisocoria, encephalopathy	CT brain: bilateral small enhancing lesions in anterior internal capsules with edema	Bilateral internal capsule abscesses	Pyrimethamine, sulfadiazine, corticosteroids	~4 weeks (stopped due to pancytopenia)	Levodopa/carbidopa and baclofen ineffective	Deteriorated; died of septicemia	4 months	Lesions disrupted nigrostriatal pathways or directly damaged striatum; postulated mechanisms include mass effect, ische

																					mia, or direct destr uction

References

1. Mohammadzadeh N, Tung GA, Prakash P, Alhusaini S. Parkinsonism: A rare complication of cerebral toxoplasmosis. Ann Neurol. 2023;94(5):853–4. doi:10.1002/ana.26808.

2. Malaquias MJ, Magrinelli F, Quattrone A, Neo RJ, Latorre A, Mulroy E, et al. Presynaptic hemiparkinsonism following cerebral toxoplasmosis: Case report and literature review. Mov Disord Clin Pract. 2023;10(2):285–99. doi:10.1002/mdc3.13631.

3. Donlon E, Theze S, Fearon C. Alternate hemibody hyperkinetic and hypokinetic movement disorders due to strategic lesions in cerebral toxoplasmosis. JAMA Neurol. 2023;80(10):1113–4. doi:10.1001/jamaneurol.2023.2059.

4. Malhotra K, Khunger M, Pu C, Scott TF. A man in his 40s with altered mental status, ataxia and unilateral weakness. Neuropathology. 2017;37(3):267–73. doi:10.1111/neup.12351.

5. Akinci Y, Sevindik MS, Mete B, Erturk UŞ, Yürüyen M, Kocazeybek BS, et al. A case of neuro-AIDS presenting with rapidly progressive dementia and parkinsonism with rapid response to treatment. Turk Noroloji Derg. 2017;23(2):81–3. doi:10.4274/tnd.94758.

6. Arbune AA, Arbune M, Stefanescu V. Parkinsonian syndrome and toxoplasmic encephalitis. J Crit Care Med (Targu Mures). 2016;2(2):89–92.

7. Murakami T, Nakajima M, Nakamura T, Hara A, Uyama E, Mita S, et al. Parkinsonian symptoms as an initial manifestation in a Japanese patient with acquired immunodeficiency syndrome and Toxoplasma infection. Intern Med. 2000;39(12):1111–4.

8. Maggi P, de Mari M, Moramarco A, Fiorentino G, Lamberti P, Angarano G. Parkinsonism in a patient with AIDS and cerebral opportunistic granulomatous lesions. Neurol Sci. 2000;21(3):173–6. DOI: 10.1007/s100720070093.

9. Carrazana EJ, Rossitch E Jr, Samuels MA. Parkinsonian symptoms in a patient with AIDS and cerebral toxoplasmosis. J Neurol Neurosurg Psychiatry. 1989;52(12):1445–1447. doi:10.1136/jnnp.52.12.1445-a.

Supplementary Table 3: Case-wise Clinical, Neuroimaging, Treatment, and Outcome Details of Ataxia and Cerebellar Syndromes in Central Nervous System Toxoplasmosis (n = 9)

Author/Year	Country	Age/Sex	Immune Status	CD4 count	Duration of illness	Mode of Infection	Diagnostic Method for Toxoplasmosis	CNS Involvement Pattern	Movement Disorder Type	Onset Relative to Infection	Duration of Movement Disorder	Severity of Movement Disorder	Other Neurological Features	Neuroimaging	Lesion Location and type	Anti-Toxoplasma Treatment	Duration of Treatment	Symptomatic Therapy for movement disorders	Outcome	Follow-up	Pathophysiological Hypothesis
Turkistani et al., 2024	Saudi Arabia	53/F	Newly diagnosed HIV	CD4 = 168 initially, 13 later	1 month	Likely reactivation of latent infection	Positive HIV antibodies, Toxoplasma IgG = 111.10 IU/mL, histopathology of cerebellar	Solitary cerebellar lesion	Ataxia	Initial manifestation	1 month	Moderate (ataxic gait, falls)	Blurred vision, headache, vomiting, ataxia	MRI: ring-enhancing lesion in left cerebellar hemisphere with edema and mass effect	Left cerebellar hemisphere mass, confirmed as Toxoplasma by histopathology	Pyrimethamine, sulfadiazine, leucovorin, ART (Biktarvy®)	2 months	Not specified	Clinical and radiological improvement	Follow-up MRI after one year showed resolution	Direct cerebellar involvement by Toxoplasma gondii causing mass effect and inflammation

							lesion							on 4th ventricle							
Asensi Cantó et al., 2023	Spain	54/F	HSCT recipient (immunocompromised)	Not reported	Subacute (~3 months post-HSCT)	Reactivation (IgG+/IgM− history; IgM+ at presentation)	PCR for T. gondii DNA in CSF and blood; MRI; muscle biopsy	Symmetric thalamic hyperintensity, cerebellitis; no ring-enhancing lesions	Cerebellar syndrome with horizontal nystagmus, dysarthria and truncal and limb ataxia	Concurrent with systemic infection	Not applicable	N/A	Proximal lower limb weakness, cerebellar syndrome: nystagmus, dysarthria, ataxia; encephalopathy	MRI: thalamic hyperintensity, cerebellitis; muscle MRI: myositis in lower limbs	Thalami and cerebellum (MRI); muscle biopsy confirmed toxoplasma cysts	Pyrimethamine + sulfadiazine (induction), corticosteroids, mycophenolate mofetil	Until negative PCR and immunosuppression taper	None specific for movement disorder	Progressive resolution of symptoms	Continued until immunosuppressive withdrawal	Toxoplasma reactivation in CNS and muscle due to immunosuppression; immune-mediated tissue injury
Gaggero et al., 2022	Italy	43/F	HIV-positive (ART interrupted for 3 years)	Not reported	Few days of headache and ataxia	Likely reactivation due to ART interruption	MRI; histology; PCR on paraffin-embedded brain tissue	Necrotizing cerebellitis with leptomeningeal involvement	Ataxia	Acute	Not applicable	Severe, rapidly progressive cerebellar syndrome	Headache, gait ataxia, dysarthria	MRI: enhancing lesion with mass effect in cerebellum;	Both cerebellar hemispheres and vermis; necrotizing	Empirical antibiotics; no specific anti-Toxoplasma therapy given	Not applicable	None given before death	Fatal (patient died rapidly after initial improvement)	No follow-up (autopsy-based diagnosis)	Toxoplasma reactivation in cerebellum with necrotizing abscess formation

														compression of 4th ventricle	lesions with Toxoplasma pseudocysts and tachyzoites	before death					due to immunosuppression from ART discontinuation
Gottlieb et al., 2020	USA	53/F	HIV-positive, untreated	20 cells/mm^3	2–3 weeks	Reactivation (latent infection)	MRI; Positive serum Toxoplasma IgG; Clinical context	Multiple ring-enhancing lesions with edema; consistent with cerebral toxoplasmosis	Ataxia and other cerebellar signs	Subacute onset	Not applicable	Moderate to severe; gait imbalance, weakness	Headache, unsteady gait, mild confusion, right-sided weakness	MRI: multiple ring-enhancing lesions in basal ganglia, thalamus, and frontal lobes	Bilateral basal ganglia, thalamus, frontal lobes	Pyrimethamine, sulfadiazine, leucovorin; ART started later	Standard course initiated	Not mentioned	Marked improvement over weeks with treatment	Follow-up reported over several weeks	Toxoplasma reactivation due to profound immunosuppression led to multifocal necrotizing encephalitis
Harbada et al.,	India (Patien	23/Mal	Immunocompetent (G6PD deficient)	Not applicable	2 weeks	Likely ingestion (G6	Histopathology (pseudoc	Cerebellum, brainstem	Ataxia	Early onset (initial	Progressive until	Severe (broad-based gait,	Scanning speech, cranial	MRI: ill-defined hyperint	Right cerebellum, brai	Clindamycin, Azithromycin	Less than 10 days (patient	Steroids	Death due to progressiv	Short (died on 10th day post-	G6PD deficiency impairs neutro

2016	t from Oman)	e				PD deficiency as a risk factor)	ysts of T. gondii), IgG positive			presentation)	death	dysmetria, dysdiadochokinesia)	nerve VI & VII palsy, nystagmus, dysgraphia, slurred speech	ense lesion with ring enhancement, perilesional edema, post-op progression	nstem; necrotic, hemorrhagic with encephalitis	(pyrimethamine-sulfadiazine avoided due to G6PD deficiency)	died)		e cerebritis and encephalitis	surgery)	phil function, reducing protozoal killing and immune control, leading to severe cerebritis with necrosis, vasculitis, and inflammation.
Soleimani & Bairami, 2015	Iran	36/M	Newly diagnosed HIV/AIDS	60 cells/μL	2 weeks	Likely reactivation in an undiagnosed AIDS pati	MRI, Toxoplasma IgG (650 IU/mL), HIV ELISA and Western	Bilateral cerebral hemispheres (ring-enhancing lesions);	Ataxia, dysarthria	Presenting feature	2 weeks	Moderate	Loss of consciousness, headache, speech impairment, imbalance	MRI: multiple ring-enhancing lesions in bilateral cerebral hem	Right posterior parietal, corpus callosum rostrum, left	IV cotrimoxazole (trimethoprim-sulfamethoxazole), dexamethason	6 weeks	Not specified	Clinical improvement: consciousness regained in 1 week;	MRI showed near-total lesion resolution by day 21; ART and cotrim	Reactivation of latent toxoplasmosis is due to advanced undiagnosed HIV infecti

						ent	blot	some cerebellar involvement						ispheres, early transtentorial herniation	anterior temporal, cerebellar hemispheres	e, antiretroviral therapy			ataxia/dysarthria resolved in 2 weeks	oxazole prophylaxis advised	on
Pott Jr. & Castelo / 2013	Brazil	50 / M	HIV-positive	43 cells/mm^3	1 week	Likely reactivation of latent infection	Serology (IgG +), MRI (ring-enhancing lesion), response to therapy	Isolated cerebellar involvement	Ataxia, wide-based gait, incoordination	Acute	Approx. 2 weeks	Moderate; resolved by discharge	Clipped speech, mild gait ataxia	MRI: ring-enhancing lesion, CT: hypodense non-enhancing cerebellar lesion	Right cerebellar hemisphere and vermis; peripheral enhancement	Sulfadiazine + Pyrimethamine + Folinic acid + corticosteroids	Not specified (improved in 2 weeks)	Not described	Complete remission of cerebellar signs	14 days (during hospital stay)	Isolated cerebellar toxoplasmosis is possibly due to atypical reactivation or neurotropism in specific site
Emeka et al., 2010	Nigeria	34 / M	HIV positive (AIDS)	Not reported	1 week (initial), recurren	Likely reactivation of latent infe	CT scan showing ring-enhancing lesio	Isolated cerebellar involvement	Ataxia, gait disturbance, dysmet	Late-stage HIV	Improved within 7–10 days of	Severe enough to impair walking and writing	Dysgraphia, right-sided adiodochokinesia,	CT scan showing ring-enhancing lesio	Right cerebellar hemisphere; ring-	Clindamycin, pyrimethamine, pyridoxine	7 days (initial), 10 days (recurrent)	Not specified	Good clinical response; relapse	Recurrent symptoms after 3 months, re-treate	Cerebellar lesion due to toxoplasmic encephalitis in

					ce after 3 months	ction	n in right cerebellar hemisphere		ria, nystagmus		treatment		slurred speech	n	enhancing lesion				due to non-compliance, responded again	d successfully	immunocompromised host
Greenlee et al., 1975	USA	60/M	Immunocompetent	Not applicable	3 years (recurrent)	Acquired	Sabin-Feldman dye test, muscle biopsy, mouse inoculation, serology	Cerebellar involvement, spinal cord, and nerve roots	Cerebellar ataxia, intention tremor	Subacute to chronic	Persistent with relapses	Moderate	Fasciculations, neuropathy, ataxia, intention tremor, hypoactive reflexes	Normal brain scan, EEG abnormalities	Histologic evidence in muscle; cerebellar and spinal features clinically	Sulfadiazine, pyrimethamine, folinic acid	Multiple courses over 3 years	Not specified	Recurrent symptoms, mild residual ataxia	3 years	Direct invasion of CNS and muscle by T. gondii; impaired lymphocyte response

AIDS = Acquired Immunodeficiency Syndrome, ART = Antiretroviral Therapy, CD4 = Cluster of Differentiation 4 (T-helper lymphocyte count), CSF = Cerebrospinal Fluid, CT = Computed Tomography, EEG = Electroencephalogram, ELISA = Enzyme-Linked Immunosorbent Assay, G6PD = Glucose-6-Phosphate Dehydrogenase, HIV = Human Immunodeficiency Virus, HSCT = Hematopoietic Stem Cell Transplantation, IgG = Immunoglobulin G, IgM = Immunoglobulin M, IV = Intravenous, MRI = Magnetic Resonance Imaging, PCR = Polymerase Chain Reaction

References

1. Turkistani AN, AlSindi T, Homoud M, Alghamdi F, Baeesa SS. Solitary cerebellar toxoplasmosis as the first presentation of HIV infection: A case report and review of literature. Cureus. 2024;16(9):e70456. doi:10.7759/cureus.70456.

2. Asensi Cantó P, Mayordomo E, Dorado A, Villalba M, Mañez RB, González E, et al. Disseminated toxoplasma infection after hematopoietic stem cell transplantation with myositis and encephalitis. Transpl Infect Dis. 2023;25(4):e14067. doi:10.1111/tid.14067.

3. Gaggero G, Campora M, Dose B, Taietti D, Vena A, Delfino E. Neuro-toxoplasmosis and fatal necrotizing cerebellitis. Autops Case Rep. 2022;12:e2021363. doi:10.4322/acr.2021.363.
4. Gottlieb GS, Rosenberg JM, González RG, Gandhi RT. Case 27-2020: A 53-year-old woman with headache and gait imbalance. N Engl J Med. 2020;383(9):859–66. doi:10.1056/NEJMcpc1913472.

5. Harbada RK, Sorabjee JS, Surya N, Jadhav KA, Mirgh S. Cerebellar toxoplasmosis in an immunocompetent patient with G6PD deficiency. J Assoc Physicians India. 2016;64(8):79–82.

6. Soleimani A, Bairami A. Cerebral toxoplasmosis in a patient leads to diagnosis of AIDS. Asian Pac J Trop Dis. 2015;5(8):667–8. doi:10.1016/S2222-1808(15)60910-0.

7. Pott H Jr, Castelo A. Isolated cerebellar toxoplasmosis as a complication of HIV infection. Int J STD AIDS. 2013;24(1):70–2. doi:10.1258/ijsa.2012.012189.

8. Emeka E, Ogunrin A, Olubunmi A. Cerebellar toxoplasmosis in HIV/AIDS: a case report. West Afr J Med. 2010;29(2):129–31.

9. Greenlee JE, Johnson WD Jr, Campa JF, Adelman LS, Sande MA. Adult toxoplasmosis presenting as polymyositis and cerebellar ataxia. Ann Intern Med. 1975;82(3):367–371. doi:10.7326/0003-4819-82-3-367.
