# Supplementary material for: Movement Disorders in Toxoplasmosis: A Systematic Review
Source: Tremor Other Hyperkinet Mov (N Y). 2025 Sep 30;15:48. doi: 10.5334/tohm.1093 (PMC12493030; doi:10.5334/tohm.1093)
Supplement: Supplementary Item 1. — Evaluation of the methodological quality of case reports and case series. [file tohm-15-1-1093-s2.pdf]

Movement Disorders in Toxoplasmosis: A Systematic Review

Supplementary item-1: Evaluation of the methodological quality of case reports and case series

Reference	Does the patient represent the whole experience of the investigator	Was the exposure adequately ascertained?	Was the outcome adequately ascertained?	Were other alternative causes that may explain the observation ruled out?	Was there a challenge and/or re-challenge phenomenon?	Was there a dose-response effect?	Was follow-up long enough for outcomes to occur?	Is the case(s) described with sufficient details to allow practitioners make inferences related to their own practice?	Score
**Hyperkinetic Movement Disorders**									
Violante-Villanueva et al., 2023	**Yes**	**Yes**	**Yes**	**Yes**	**NA**	**Yes**	**Yes**	**No**	**6**
Rocha-Cadman et al., 2024	**Yes**	**Yes**	**Yes**	**Yes**	**NA**	**Yes**	**Yes**	**Yes**	**7**
Önder, 2023	**Yes**	**Yes**	**Yes**	**Yes**	**NA**	**Yes**	**Yes**	**Yes**	**7**
Dimal et al., 2021	**Yes**	**Yes**	**Yes**	**Yes**	**NA**	**Yes**	**Yes**	**Yes**	**7**
Rissardo et al., 2020	**Yes**	**Yes**	**Yes**	**Yes**	**NA**	**Yes**	**Yes**	**No**	**7**
Reyes et al., 2018	**Yes**	**Yes**	**Yes**	**Yes**	**NA**	**Yes**	**Yes**	**No**	**7**
AbdelRazek & Venna, 2018	**Yes**	**Yes**	**Yes**	**Yes**	**NA**	**No**	**No**	**Yes**	**5**
Cambrea et al.,	**Yes**	**Yes**	**Yes**	**Yes**	**NA**	**Yes**	**Yes**	**Yes**	**7**

2017									
Reyes et al., 2016	**Yes**	**Yes**	**Yes**	**Yes**	**NA**	**Yes**	**Yes**	**Yes**	**7**
Berlot et al., 2015	**Yes**	**Yes**	**Yes**	**Yes**	**NA**	**Yes**	**Yes**	**Yes**	**7**
Wu et al., 2014	**Yes**	**Yes**	**Yes**	**Yes**	**NA**	**Yes**	**Yes**	**Yes**	**7**
Mishra et al., 2014	**Yes**	**Yes**	**Yes**	**Yes**	**NA**	**Yes**	**Yes**	**Yes**	**7**
	**Yes**	**Yes**	**Yes**	**Yes**	**NA**	**Yes**	**Yes**	**Yes**	**7**
Moccia et al., 2013	**Yes**	**Yes**	**Yes**	**Yes**	**NA**	**Yes**	**Yes**	**Yes**	**7**
	**Yes**	**Yes**	**Yes**	**Yes**	**NA**	**Yes**	**Yes**	**Yes**	**7**
Rabhi et al., 2011	**Yes**	**Yes**	**Yes**	**Yes**	**NA**	**Yes**	**Yes**	**Yes**	**7**
Lekoubou et al., 2010	**Yes**	**Yes**	**Yes**	**Yes**	**NA**	**Yes**	**Yes**	**Yes**	**7**
Henriques Aquino et al., 2010	**Yes**	**Yes**	**Yes**	**Yes**	**NA**	**Yes**	**Yes**	**Yes**	**7**
Midi et al., 2008	**Yes**	**Yes**	**Yes**	**Yes**	**NA**	**Yes**	**Yes**	**Yes**	**7**
Zuniga et al., 2005	**Yes**	**Yes**	**Yes**	**Yes**	**NA**	**Yes**	**Yes**	**Yes**	**7**
Factor et al., 2003	**Yes**	**Yes**	**Yes**	**Yes**	**NA**	**Yes**	**Yes**	**Yes**	**7**
Pezzini et al., 2002	**Yes**	**Yes**	**Yes**	**Yes**	**NA**	**Yes**	**Yes**	**Yes**	**7**
Piccolo et al., 1999	**Yes**	**Yes**	**Yes**	**Yes**	**NA**	**Yes**	**Yes**	**Yes**	**7**
	**Yes**	**Yes**	**Yes**	**Yes**	**NA**	**Yes**	**Yes**	**Yes**	**7**
	**Yes**	**Yes**	**Yes**	**Yes**	**NA**	**Yes**	**Yes**	**Yes**	**7**
	**Yes**	**Yes**	**Yes**	**Yes**	**NA**	**Yes**	**Yes**	**Yes**	**7**

	**Yes**	**Yes**	**Yes**	**Yes**	**NA**	**Yes**	**Yes**	**Yes**	**7**
Micheli et al., 1997	**Yes**	**Yes**	**Yes**	**Yes**	**NA**	**Yes**	**Yes**	**Yes**	**7**
Maher et al., 1997	**Yes**	**Yes**	**Yes**	**Yes**	**NA**	**Yes**	**Yes**	**Yes**	**7**
Maggi et al., 1996	**Yes**	**Yes**	**Yes**	**Yes**	**NA**	**Yes**	**Yes**	**Yes**	**7**
	**Yes**	**Yes**	**Yes**	**Yes**	**NA**	**Yes**	**Yes**	**Yes**	**7**
	**Yes**	**Yes**	**Yes**	**Yes**	**NA**	**Yes**	**Yes**	**Yes**	**7**
Garretto et al., 1995	**Yes**	**Yes**	**Yes**	**Yes**	**NA**	**Yes**	**Yes**	**Yes**	**7**
Tedrus et al., 1994	**Yes**	**Yes**	**Yes**	**Yes**	**NA**	**Yes**	**Yes**	**Yes**	**7**
Micheli et al., 1994	**Yes**	**Yes**	**Yes**	**Yes**	**NA**	**Yes**	**Yes**	**Yes**	**7**
Nath et al., 1993	**Yes**	**Yes**	**Yes**	**Yes**	**NA**	**Yes**	**Yes**	**Yes**	**7**
de Mattos et al., 1993	**Yes**	**Yes**	**Yes**	**Yes**	**NA**	**Yes**	**Yes**	**Yes**	**7**
	**Yes**	**Yes**	**Yes**	**Yes**	**NA**	**Yes**	**Yes**	**Yes**	**7**
Tolge & Factor / 1991	**Yes**	**Yes**	**Yes**	**Yes**	**NA**	**Yes**	**Yes**	**Yes**	**7**
Koppel & Daras, 1990	**Yes**	**Yes**	**Yes**	**Yes**	**NA**	**Yes**	**Yes**	**Yes**	**7**
Sanchez-Ramos et al., 1989	**Yes**	**Yes**	**Yes**	**Yes**	**NA**	**Yes**	**Yes**	**Yes**	**7**
Carrazana et al., 1989	**Yes**	**Yes**	**Yes**	**Yes**	**NA**	**Yes**	**Yes**	**Yes**	**7**
**Hypokinetic Movement Disorders**									
Mohammadzadeh et al., 2023	**Yes**	**Yes**	**Yes**	**Yes**	**NA**	**Yes**	**Yes**	**Yes**	**7**
Malaquias et al., 2023	**Yes**	**Yes**	**Yes**	**Yes**	**NA**	**Yes**	**Yes**	**Yes**	**7**
Donlon et al.,	**Yes**	**Yes**	**Yes**	**Yes**	**NA**	**Yes**	**Yes**	**Yes**	**7**

2023									
Malhotra et al., 2017	**Yes**	**Yes**	**Yes**	**Yes**	**NA**	**Yes**	**Yes**	**Yes**	**7**
Akinci et al., 2017	**Yes**	**Yes**	**Yes**	**Yes**	**NA**	**Yes**	**Yes**	**Yes**	**7**
Arbune et al., 2016	**Yes**	**Yes**	**Yes**	**Yes**	**NA**	**Yes**	**Yes**	**Yes**	**7**
Murakami et al., 2000	**Yes**	**Yes**	**Yes**	**Yes**	**NA**	**Yes**	**Yes**	**Yes**	**7**
Maggi et al., 2000	**Yes**	**Yes**	**Yes**	**Yes**	**NA**	**Yes**	**Yes**	**Yes**	**7**
Carrazana et al., 1989	**Yes**	**Yes**	**Yes**	**Yes**	**NA**	**Yes**	**Yes**	**Yes**	**7**
**Ataxia and Cerebellar Syndromes**									
Turkistani et al., 2024	**Yes**	**Yes**	**Yes**	**Yes**	**NA**	**Yes**	**Yes**	**Yes**	**7**
Asensi Cantó et al., 2023	**Yes**	**Yes**	**Yes**	**Yes**	**NA**	**Yes**	**Yes**	**Yes**	**7**
Gaggero et al., 2022	**Yes**	**Yes**	**Yes**	**Yes**	**NA**	**Yes**	**Yes**	**Yes**	**7**
Gottlieb et al., 2020	**Yes**	**Yes**	**Yes**	**Yes**	**NA**	**Yes**	**Yes**	**Yes**	**7**
Harbada et al., 2016	**Yes**	**Yes**	**Yes**	**Yes**	**NA**	**Yes**	**Yes**	**Yes**	**7**
Soleimani & Bairami, 2015	**Yes**	**Yes**	**Yes**	**Yes**	**NA**	**Yes**	**Yes**	**Yes**	**7**
Pott Jr. & Castelo / 2013	**Yes**	**Yes**	**Yes**	**Yes**	**NA**	**Yes**	**Yes**	**Yes**	**7**
Emeka et al., 2010	**Yes**	**Yes**	**Yes**	**Yes**	**NA**	**Yes**	**Yes**	**Yes**	**7**
Greenlee et al., 1975	**Yes**	**Yes**	**Yes**	**Yes**	**NA**	**Yes**	**Yes**	**Yes**	**7**

All 60 cases are of good quality.

Domains Leading explanatory questions
Selection 1. Does the patient(s) represent(s) the whole experience of the investigator or is the selection method unclear to the extent that other patients with similar presentation may not have been reported?

Ascertainment 2. Was the exposure adequately ascertained?

3. Was the outcome adequately ascertained?
Causality
4. Were other alternative causes that may explain the observation ruled out?
5. Was there a challenge/re-challenge phenomenon?
6. Was there a dose–response effect?
7. Was follow-up long enough for outcomes to occur?

Reporting
8. Is the case(s) described with sufficient details to allow other investigators to replicate the research or to allow practitioners make inferences related to their own practice?
